# Suspect and Nontarget Screening of Organic Micropollutants in Swiss Sewage Sludge: A Nationwide Survey

**DOI:** 10.1021/acs.est.4c13217

**Published:** 2025-04-08

**Authors:** Pablo A. Lara-Martín, Lena Schinkel, Yves Eberhard, Walter Giger, Michael Berg, Juliane Hollender

**Affiliations:** † Department of Physical Chemistry, Institute of Marine Research (INMAR), International Campus of Excellence of the Sea (CEIMAR), Faculty of Marine and Environmental Sciences, University of Cadiz, Puerto Real 11510, Spain; ‡ Eawag, Swiss Federal Institute of Aquatic Science and Technology, 8600 Dübendorf, Switzerland; § Giger Research Consulting, 8049 Zürich, Switzerland; ∥ Institute of Biogeochemistry and Pollutant Dynamics (IBP), ETH Zurich, 8092 Zurich, Switzerland

**Keywords:** sewage sludge, nontarget screening, mass spectrometry, anaerobic digestion, emerging pollutants, Kendrick mass defect

## Abstract

The increasing amount of sewage sludge generated during wastewater treatment poses both growing management challenge and environmental issues. Sludge with many co-occurring contaminants is often destined to land application which raises concern regarding human and environmental health. It is also a good integrator in time and space and can provide valuable information on consumption pattern and change over time. Here, we have conducted suspect and nontarget screening (SNTS) in sludge from 29 wastewater treatment plants (WWTPs) covering 30% of the Swiss population. Over 500 contaminants were identified and up to 382 quantified, with concentrations ranging from a few ng/g to several thousand ng/g, which translated into total annual loads of approximately 5 g of micropollutants per Swiss citizen. The distribution of detected substances was dominated by pharmaceuticals in terms of number of compounds (>250) and personal care products in terms of concentration (e.g., 75 μg/g for linoleic acid). Homologous series analysis revealed the presence of multiple classes of surfactants among those compounds with the highest signal intensities in sludge. Principal component analysis and hierarchical clustering showed that spatial distribution of contaminants across Switzerland was not homogeneous, while Pearson correlation indicated that changes can be attributed to different anaerobic digestion times in WWTPs.

## Graphical abstract

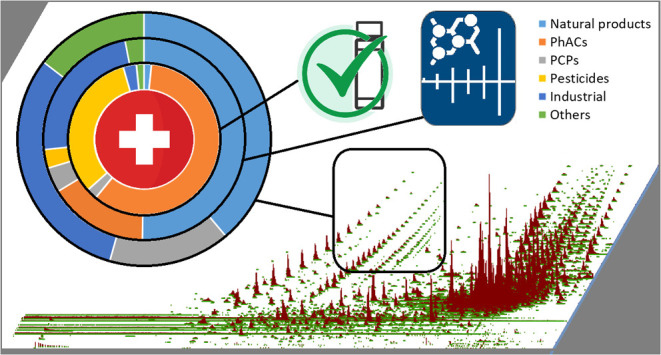

## Introduction

Sewage sludge or biosolids are generated in wastewater treatment plants (WWTPs) through primary and secondary treatment processes. Nowadays, approximately 11 million tons of sewage sludge dry matter per year are generated in the European Union (EU), and in the future, this number is expected to increase to 15 million tons.^1^ According to estimates, almost 50% of sewage sludge produced in EU countries is used for agricultural purposes, while about 12% is composted and used in the fertilization of forests and gardens.^1^ Land disposal of sludge is a controversial practice because, although rich in nutrients, biosolids can also contain pathogens, heavy metals, and organic chemicals.^2^ Only in a few countries such as Switzerland (with an annual sludge production of 0.30 million tons) land application is banned due to environmental concerns, and incineration is performed instead.^3^

Organic chemicals accumulating in sludge are compounds used in everyday products for personal care, health care, and other household purposes, as well as industrial and agrochemicals depending on the region.^4^ Concentrations of these chemicals depend on several factors, including production volume in commerce, the fraction disposed into wastewater unchanged or as transformation products (TPs), and their partitioning behavior and degradability in WWTPs. Consistent information on the level of contamination of sludge is critical for better understanding the fate of chemicals during wastewater treatment and for developing guidelines on sludge disposal and reutilization.^2^ Moreover, data on concentration of specific substances in sludge, gathered through local or national surveys, can allow to identify persistent and potentially bioaccumulative compounds that may represent contaminants of emerging concern (CECs) in the communities served by the respective WWTP.^5^

Until the past decade, most of the studies on organic contaminants in sludge have focused on specific classes of compounds. Information of their concentrations and occurrence has been compiled in several recent reviews,^1,2,6–9^ but this represents only a glimpse of the potentially thousands of synthetic chemicals occurring in sludge. A complete characterization of the organic fraction of sludge would be an extremely challenging task due to the complexity of the matrix (dominated by humic acids, biomolecules, lipids, etc.)^10^ and the enormous size of current chemical inventories, with hundreds of thousands of substances currently on the market.^11^

New analytical workflows based on high-resolution mass spectrometry (HRMS) for the suspect and nontarget screening (SNTS) of organic compounds have arisen as a very powerful tool for massive identification of known and new emerging contaminants, as well as their TPs in all kinds of environmental samples.^12^ One of the first studies, performed in Switzerland a decade ago, revealed corrosion inhibitors, artificial sweeteners, and pharmaceuticals exhibiting the highest concentrations in wastewater, as well as managed to identify sulfur-containing surfactants and a vulcanization accelerator (1,3-benzothiazole-2-sulfonate).^13^ Application of SNTS to sewage sludge is still limited although some recent studies have shed qualitatively and occasionally quantitatively some light on the hundreds of organic contaminants in this matrix.^14–19^

Here, we performed SNTS on sludge samples from 29 WWTPs in Switzerland. With this, we present a follow-up study of a previous national survey aimed to quantify the concentrations of major, trace, and rare elements in WWTPs and major rivers in the country,^3,20,21^ as well as synthetic surfactants.^22^ As sewage sludge is a good integrator in time and space, the acquired HRMS chemical fingerprint is representative of an industrialized country and can provide valuable information on consumption patterns by its population. Such data set was explored to (i) identify individual organic contaminants and homologous series that are predominant in sludge, (ii) analyze the impact of different extraction methods on such identification in the complex matrix, (iii) calculate the per capita contaminant fluxes to the sludge, and (iv) visualize and understand contaminant distribution patterns across Swiss WWTPs.

## Materials and Methods

### Sampling Campaign

Samples of digested sewage sludge (*n* = 31) were collected from 29 Swiss WWTPs between 2017 and 2020. Samples from Samedan WWTP (SAM and SAM2) were collected at two different years (2019 and 2020) and those from Münchwilen (MUEN and MUEN2) are replicates. All Swiss WWTPs were equipped with biological treatment including phosphorus elimination, nitrification, and/or denitrification, having a sludge production from 150 to 36,250 kg/day and performing anaerobic digestion between 0 and 7 weeks (average sludge age = 27 days). The sampled WWTPs were selected to guarantee a broad nationwide representation on the basis of geographical distribution, numbers of connected people, and industries in the catchment areas. Both rural and urban areas were included, representing a total of approximately 2.5 million connected inhabitants (roughly 30% of the country, with 8.7 million inhabitants in 2021). Detailed information on each sample is compiled in Table S1 in the Supporting Information (SI), including a map with the location of WWTPs and their catchments (Figure S1). Sludge samples were freeze-dried after collection and preserved at −20 °C in tightly sealed vials until extraction.

### Sample Extraction

Sample aliquots (0.2 g of freeze-dried sludge) were mixed with Hydromatrix (Thermo) and placed inside stainless steel cells in an accelerated solvent extractor (Dionex ASE 350). The instrument was operated at 100 °C and 1500 psi, with 3 extraction cycles of 5 min each and a flush volume of 60%. Two different solvents, methanol (MeOH) and water acidified with citric acid (CA), and sorbents, octadecyl silica (C18) and Florisil, were used. Three different extraction methods (MeOH/CA, MeOH + C18, and MeOH + Florisil) based on the existing literature^23–25^ were tested. The first method (MeOH/CA) used 50/50 (v/v) methanol/water (acidified at pH = 3 with citric acid) as solvent, whereas the other two used methanol only. Cleanup of the extracts was conducted either offline by solid phase extraction Bond Elut C18 cartridges (method MeOH + C18) or online (by placing 1 g of Florisil inside the ASE cells) (method MeOH + Florisil). Final volumes were adjusted to 20 mL and sample extracts were kept at −20 °C until further analysis. All extractions were performed in the same batch.

### Suspect and Nontarget Screening of Contaminants

Sample extract aliquots were diluted 100 times with 50/50 MeOH/water (v/v) and spiked with a mixture of 255 internal standards (Table S2). All samples were injected within the same sequence (5 μL per sample) on a Thermo Ultimate HPG 3400 LC system coupled to PAL RTC autosampler and a Thermo Orbitrap Exploris 240 high-resolution mass spectrometer equipped with an electrospray ionization source (ESI). Mobile phases consisted of methanol (A) and HPLC water (B), both modified either with 0.1% formic acid and 5 mM ammonium formate (for ESI+) or 5 mM ammonia and 5 mM ammonium acetate (for ESI−). Separation was performed on an Acquity UPLC BEH C18 column (1.7 μm particle size, 2.1 mm × 150 mm) from Waters using the following gradient (flow = 0.3 mL min^–1^): 5% of A for 1 min, then linearly increased to 100% of A over the next 9 min, and held at these conditions for another 3 min. Initial conditions (5% of A) were achieved in 1.5 min. The total run time was 14.5 min.

MS settings were as follows: ion source at 3.5 kV in ESI+ and 2.5 kV in ESI–, sheath gas at 40, auxiliary gas at 10, sweep gas at 0, ion transfer tube temperature at 320 °C, vaporizer temperature at 40 °C, and RF lens at 70%. Internal mass calibration was performed using RunStart EASY-IC. The mass resolution was set at 120,000 within a scan range (*m*/*z*) from 100 to 1000 for using full-scan acquisition (maximum injection time set at 100 ms). Data-dependent acquisition (DDA) was triggered in automatic range mode in the same runs using three HCD collision energies (15, 45, and 90%), a maximum injection time of 50 ms per scan, and a mass resolution of 15,000. Samples were reinjected up to 5 times using the AcquireX function to maximize the number of MS/MS spectra acquired. The total cycle time was 0.6 s.

Compound Discoverer 3.3 was used to gather mass spectrometric (MS) features and conduct suspect and nontarget identifications of organic contaminants in sludge extracts. The workflow was adapted from that previously developed for water samples^26^ (Figure S2). A combination of molecular formula prediction, online databases (ChemSpider), suspect lists of contaminants (NORMAN Substance Database 2022, >100,000 compounds), in silico fragmentation algorithms (Fragment Ion Search, or FISh, and mzLogic), and mass spectra libraries (mzCloud and MassBank EU, >30,000 and >15,000 compounds, respectively) was used to tentatively identify compounds^27^ at Levels 4–2. Prioritization of substances of interest for further identification was performed using different statistical tools (e.g., Pearson correlation analysis, principal component analysis, or PCA, and hierarchical cluster analysis, or HCA) and Kendrick mass defect plots (for detection of homologous series). Further details on the workflow used are presented in SI.

Identification at Level 1 and quantification was achieved through the use of 713 standards (Table S3), which were injected under the same conditions as the samples at 5 concentration levels (0.1–1000 μg/L). Concentrations were corrected using the corresponding internal standards when available, and later used to calculate contaminant weighted mean (WM) concentrations (in μg/kg)^22^

$$WM=\frac{\sum_{n}\left(c_{i}\times V_{i}\right)}{\sum_{n}V_{i}}$$

where *c_i_* = measured concentration and *V_i_* = daily stabilized sludge production for each WWTP. Yearly loads (kg/year) were then determined by multiplying WM by the annual Swiss sludge production (0.3 million tons per year)^3^

yearlyloads=WM×0.3

Yearly per capita mass loads (μg/year/person) were calculated by multiplying WM by the annual average per capita sludge production from all WWTPs monitored

$$\mathrm{yearly}\;\mathrm{per}\;\mathrm{capita}\;\mathrm{load}=\mathrm{WM}\times \frac{\sum_{n}V_{i}}{\sum_{n}P_{i}}\times 365$$

where *P_i_* = connected population to each WWTP.

## Results and Discussion

### Influence of the Extraction Method on the Suspect and Nontarget Screening

Figure 1A shows the number of observed full-scan MS features detected in all 31 sludge samples collected from Swiss WWTPs after performing three different extractions (MeOH/CA, MeOH + C18, and MeOH + Florisil). Over half a million features (20% in ESI– and 80% in ESI+) were observed, and 64 and 29% retrieved at least one hit or more from ChemSpider and NORMAN databases, respectively. Further identification of these features relied on obtaining MS/MS spectra, which was possible only for 24% of them even after several injections (*n* = 5) using the AcquireX function. Improving this number might not be possible unless sample concentration factor is increased due to the low MS signal (in the order of 10^3^ counts) of many features, which hampers acquiring reliable MS/MS spectra. The identification of possible contaminants (and other naturally occurring substances) in sludge was further complicated by the relatively limited number of compounds (yet) registered in HRMS spectral libraries. MzCloud and MassBank EU search retrieved 6617 and 1251 hits, respectively, with matching score >70%, which represents less than 1% of the acquired features. A more restrictive score threshold (>95%) lowered the number of hits even further down to 199 compounds identified at Level 2 (Table S4). Further confirmation of spectral library hits using available reference standards (Level 1) yielded a total of 382 contaminants in Swiss sludge (Table S5), roughly 0.1% of the total number of observed full-scan MS features.

**Figure 1:**
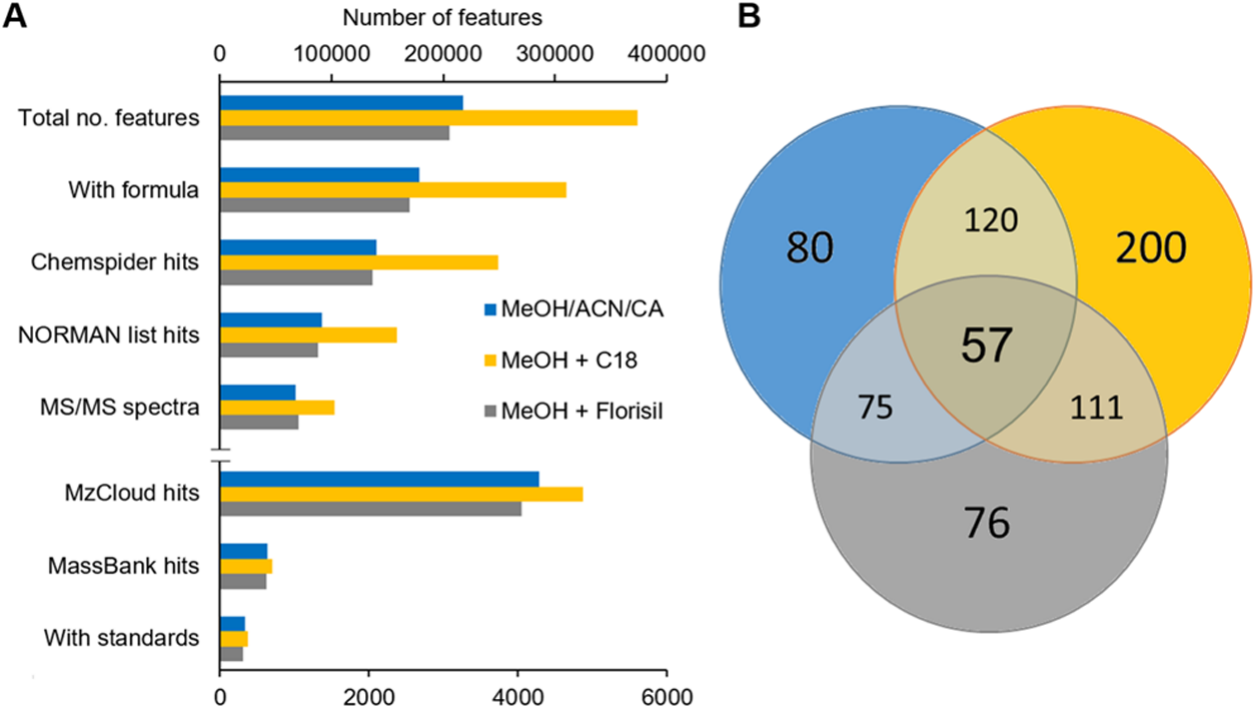
(A) Number of features (combining ESI positive and negative ionization modes) detected in sewage sludge and their annotation sources. (B) Venn diagram representing all features (in thousands) in common and different among the three extraction methods used (MeOH/CA, MeOH + C18, and MeOH + Florisil).

Extraction from sludge also affected the number of features acquired and later identified.^10^ Among the three different tested methods, MeOH + C18 resulted in the highest number of features detected (374,118) and tentatively identified by spectral libraries (4880) and reference standards (331). Features that were observed in all three different extracts represented only about 10% of the total, which exemplifies how sample preparations used covered different chemical spaces (Figure 1B). This is also illustrated in Figure S3, where the number of features extracted as a function of their chromatographic retention time is depicted. The first method used (MeOH/CA) relies on acidic water and methanol for extraction of sulfonamides, macrolides, and other pharmaceuticals,^23^ with no further cleanup. As such, more polar chemicals were extracted compared to the other two methods, resulting in over 10,000 features detected within the first 3 min in the chromatograms. On the other hand, the third method (MeOH + Florisil) uses in-cell cleanup with Florisil as a sorbent, which is suited to adsorb chemical species with low to medium polarity^24^ and resulted in >75% of the extracted features eluting within the second half of the chromatograms (retention time >6 min). The addition of Florisil also minimized signal suppression due to matrix effects, resulting in cleaner sample extracts (Figure S4). Lastly, the MeOH + C18 method sat between the other two, with the highest number of features eluting in the middle of the chromatogram. It includes an additional step after PLE involving SPE purification with C18 cartridges and was originally designed for analysis of surfactants and their metabolites in solid matrices.^25^ By covering different chemical spaces, the number of compounds identified later on at Levels 1 and 2 varied depending on the method used (between 286 and 331, and 178 and 181, respectively). Examples of contaminants that were only detected using a specific extraction method are octylphosphonic acid (method MeOH/CA) (Level 2), an industrial chemical; mepivacaine (method MeOH + C18) (Level 1), an anesthetic; and 7-aminoflunitrazepam (method MeOH + Florisil) (Level 1), a transformation product from the benzodiazepine flunitrazepam.

### Homologous Series Detection and Identification in Sludge Samples

Identification of features was extended by analysis of Kendrick mass defect plots (Figure S5). Over a hundred different homologous series were observed to occur in sludge samples (Table S6). Components from each series were identified at Levels 4–2 by using a combination of elemental composition assignment, retention time increasing as a function of the number of repeating units (CH_2_, CF_2_, C_2_H_4_O, and C_3_H_6_O were considered), spectral library search, and comparison of MS spectra with in silico fragmentation of possible candidates from compound databases. For more complex homologous series (those containing dozens of components with more than one associated feature per component), Pearson correlation analysis was performed to discern whether a component belonged to a specific series or not. This is needed as it is not uncommon that many homologues are not just a single compound (or chromatographic peak) but a mixture of isomers, including linear, mono- and multibranched alkyl chains (e.g., nonionic surfactants alcohol ethoxylates, or AEOs), as well as unsaturated carbon–carbon bonds at different positions in such alkyl chains (e.g., fatty acids). Figure S6 shows, as an example, the correlation heatmap for the homologous series tentatively identified as C8 to C42 alkyl dicarboxylic acids (ADAs), where some components (e.g., dodecanedioic acid) could be annotated using spectral libraries.

Two more examples of identified homologous series are illustrated in Figure 2, showing perfluoroalkyl carboxylic acids (PFCAs) having between 4 and 13 C atoms and alkyl glycol ether sulfates (PEG-SO_3_) with 2–11 ethoxylated units (EO). The first have previously been reported, together with perfluoroalkyl sulfonic acids (PFSAs), in effluent wastewater from Swiss WWTPs,^28^ with those with 8 C atoms (PFOA) being predominant in the dissolved phase, whereas PFHxA (C6) and those with longer alkyl chains (>10) were present at higher signal intensities in our sludge samples. Another sampling campaign conducted in 2011 revealed some elevated PFCA levels in Swiss sludge (up to 233 ng/g) attributed to emissions from textile finishing industries.^29^ A more comprehensive study focused on per- and polyfluoroalkyl substances (PFAS) was recently conducted in France, where a combination of target and nontarget screening revealed up to 160 of such chemicals, including newly marketed zwitterionic and cationic PFAS, occurring in sewage sludge and other organic waste products for land application.^16^

**Figure 2:**
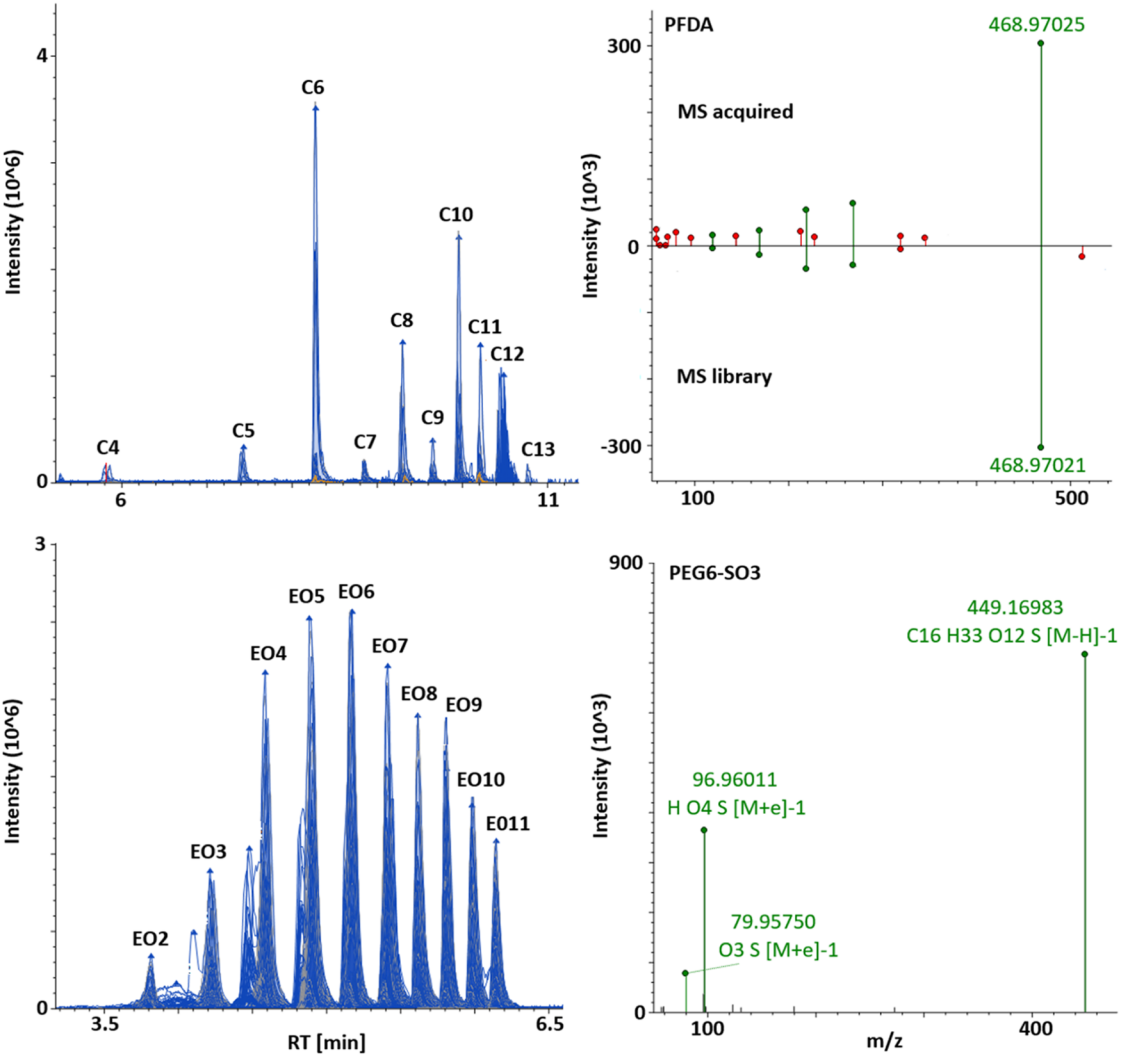
Examples of identification at Level 2 (perfluoroalkyl carboxylic acids, PFCAs) (top) and Level 3 (alkyl glycol ether sulfates, PEG-SO_3_) (bottom) of homologous series in sewage sludge: chromatograms (left) and mass spectrum annotations for perfluorodecanoic acid (PFDA) by MS library and hexaglycol ether sulfate (PEG6-SO_3_) by Fragment Ion Search (FISh) (right).

Alkyl glycol ether sulfates, the second homologous series in Figure 2 are anionic surfactants never identified before in environmental samples until 2015, when NTS was applied to influent and effluent sewage samples from Greek WWTPs.^30^ Such compounds, used in personal care products (PCPs) and transformation products (TPs) of other anionic surfactants, exhibited among the highest intensities in the ESI– mode in wastewater. An additional retrospective suspect screening of surfactants using a list of 398 chemicals was also performed in that work, reporting the occurrence of 110 suspects (e.g., linear alkylbenzenesulfonates, LAS, sulfophenyl carboxylic acids, SPCs, and alcohol ethoxylates, AEOs, among others). In our case, we conducted a more detailed analysis using a more comprehensive suspect list in combination with Kendrick mass defect plots. This approach resulted in the annotation of 37 out of 103 homologous series as anionic (*n* = 10), nonionic (*n* = 21), and cationic (*n* = 6) surfactants used in industrial and/or household applications (Table S6). A few industrial chemicals could also be identified, including flame retardants and/or plasticizers (bisphenols, phthalates, and alkyl/phenyl phosphates), and the aforementioned PFAS. The remaining homologous series (>50) were tentatively annotated as naturally occurring products (mostly fatty acids) and were not further explored as their identification with higher level of confidence goes beyond the scope of this work. It is noteworthy to point out, however, that many of these compounds are not only cell components but are also extracted/synthesized by industry to be used in the formulation of hygiene and personal care products among other applications. To discern the relative contributions of both natural and anthropogenic sources is challenging and certainly would not be possible without using additional analytical techniques (e.g., compound-specific stable isotope analysis).^26^

Annotation of features in SNTS by considering their belonging to a homologous series adds another layer of information to those from more commonly used sources (e.g., mass spectral libraries and lists of suspect compounds). In our case, 103 homologous series (comprising over 1600 individual components altogether) were added to the 581 contaminants already identified at Levels 1 and 2 (mainly pharmaceuticals and pesticides), thus increasing the number of identified natural products, industrial chemicals, and PCPs (Figure S7), for which analytical standards often might not be available. In fact, only 3 compounds (linoleic acid, octocrylene, and tramadol) out of the top 100 could be identified in our sludge samples by using reference standards. Homologous series analysis is thus a very valuable tool when trying to annotate high-intensity features in complex and heavily polluted environmental matrices, namely, wastewater and sewage sludge (Figure 3), which are disposed to the environment through effluent discharge and land application, respectively.^16^

**Figure 3:**
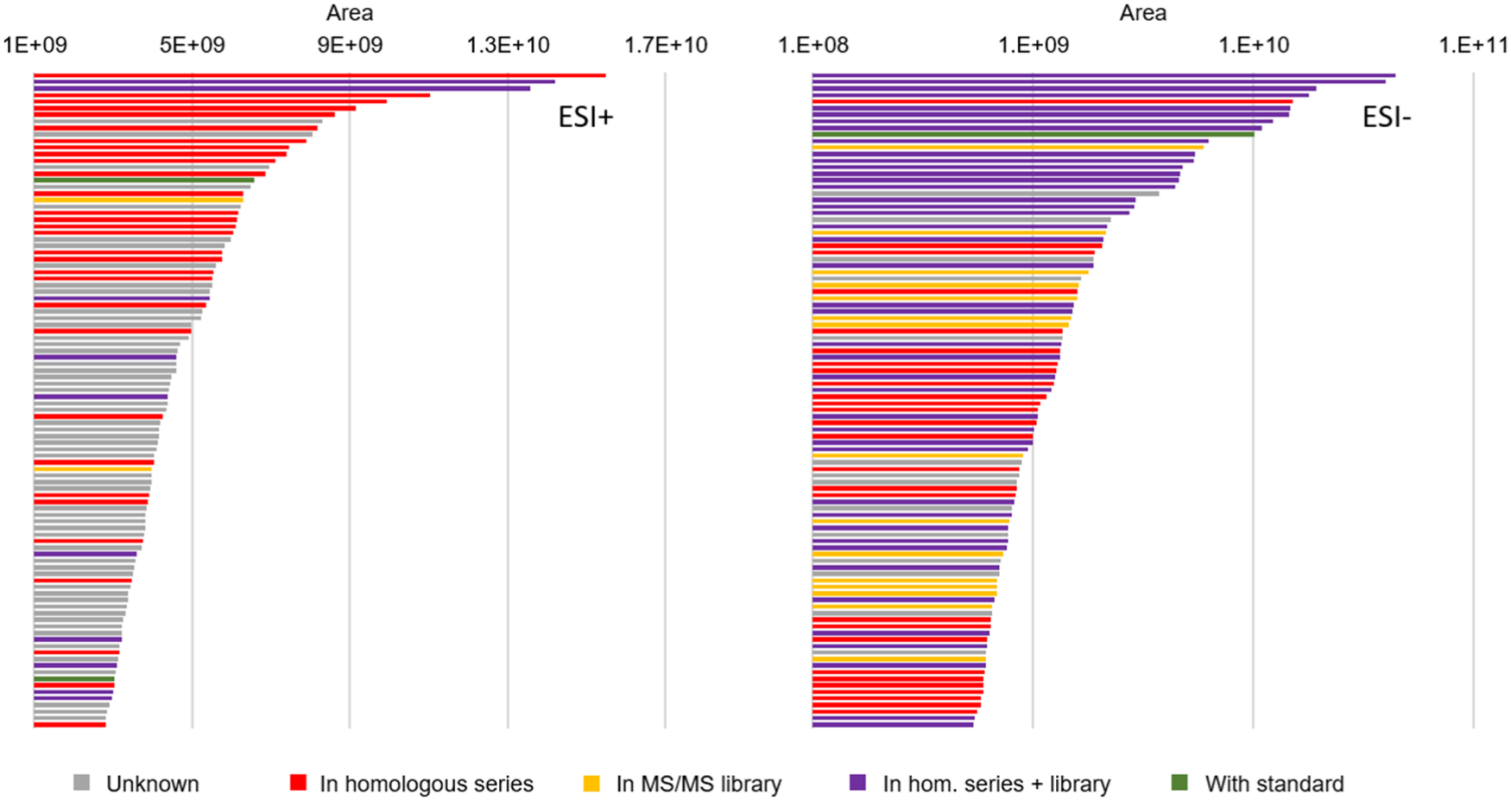
Top ESI± 100 features in sewage sludge samples regarding the peak area for which components were annotated by reference standards, MS/MS libraries, and/or homologous series analysis.

### Occurrence and Loads of Contaminants in Switzerland

Figure 4 shows the concentration ranges (A) and loads (B) of the most abundant organic contaminants (with both frequency of detection >50% and mean concentration >100 ng/g) in Swiss sewage sludge that were identified at Level 1 and quantified. Most of them belong to the pharmaceutical class (*n* = 25). Levofloxacin (fluoroquinolone antibiotic), clotrimazole (antifungal), tetrahydrocannabinol (THC, psychoactive ingredient in cannabis), bufexamac (anti-inflammatory), and telmirsartan (antihypertensive), detected in >90% of the samples at mean concentrations between 600 and 1900 ng/g, were the predominant pharmaceutically active compounds (PhACs) in sludge. They serve to illustrate the enormous variety within this class of chemicals occurring in the environment. Over 250 different PhACs were identified in sewage sludge at Levels 1 (88%) and 2 (22%). They cover more than 20 PhAC subclasses, most frequently psychiatric drugs (42), antibiotics (17), illicit/recreational drugs (15), anti-inflammatories (13), and pain relievers (10), as well as their TPs (51). The presence of PhACs in WWTPs has been exhaustively investigated over the past two decades, with the earliest works in Switzerland reporting antibiotics (sulfapyridine, sulfamethoxazole, trimethoprim, azithromycin, and clarithromycin) in conventional activated sludge treatment at concentrations between 28 and 68 ng/g of dry weight.^31^ Nowadays, fluoroquinolones and biocides currently dominate this PhAC subclass, with mean concentrations of 6500 and 2300 ng/g, respectively.^32^

**Figure 4:**
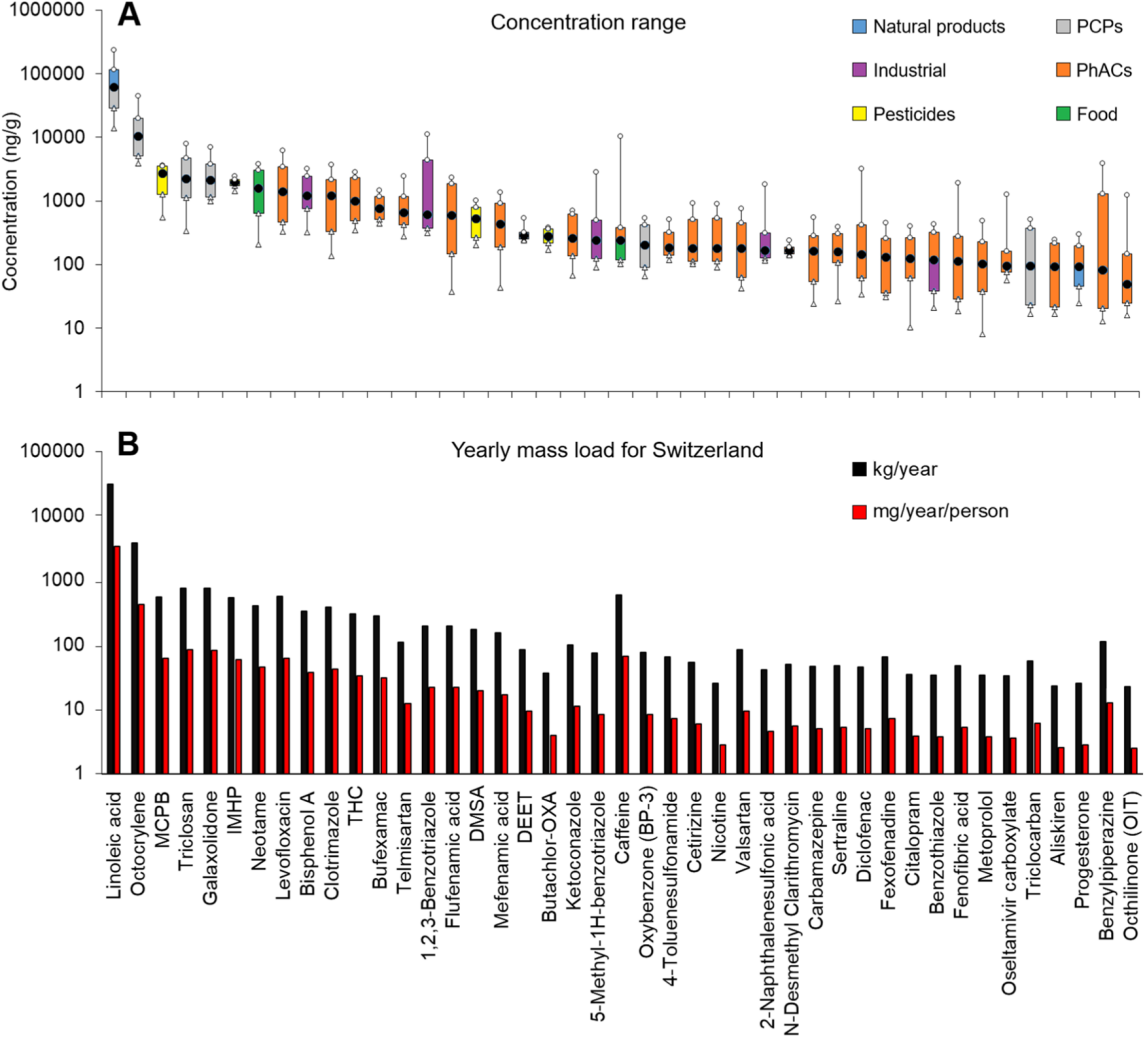
(A) Concentration ranges (ng/g) of most frequently detected organic contaminants (identified at Level 1) in sludge samples from 29 Swiss WWTPs. (B) Yearly mass load for Switzerland (kg/year) and per capital load (mg/year/person) are also presented.

The most recent works on STNS were performed on sewage sludge samples from five different WWTPs in Denmark^17^ and 3 WWTPs in Nigeria,^19^ and include semiquantification after nontarget screening. In the first one, 120 micropollutants were discriminated against over 21,000 features, of which pharmaceuticals contributed the largest share followed by pesticides and natural products. Concentrations of identified contaminants ranged between 0.2 and 10,881 ng/g dry matter. In the second work, 250 compounds were identified (182 also quantified), with pharmaceuticals being predominant, including salicylic acid occurring at the highest concentration (72,400 ng/g) and the antibiotics ciprofloxacin and ofloxacin up to 24,400 and 28,400 ng/g, respectively. These numbers are key to refine calculations in prescription^33^ and illicit drug consumption^34^ when applying wastewater-based epidemiology (WBE), which often relies on the analysis of the dissolved fraction of wastewater only. This applies to, e.g., cannabinoids, with higher lipophilicity compared to other drugs, or the cationic β-blockers, which, in spite of their low *K*_ow_ values, exhibit strong sorption dominated by electrostatic interaction. Our data indicate that the contribution of THC and metoprolol to sludge in Switzerland involves 300 kg/year and 33 mg/year/person and 34 kg/year and 4 mg/year/person, respectively.

Although not dominant by number of compounds identified, the highest concentrations (>1000 ng/g) measured in sludge belonged to ingredients used in PCPs: emollient and thickening agents (linoleic acid), UV filters (octocrylene, OC), antimicrobials (triclosan, TCS), and synthetic fragrances (galaxolidone, a transformation product of galaxolide). Organic UV filters have been reported in WWTP influents in previous sampling campaigns in Switzerland,^35^ where four of the most important compounds (benzophenone-3, BP-3; 4-methylbenzylidene camphor, 4-MBC; ethylhexyl methoxy cinnamate, EHMC; and OC) were detected at concentrations of several μg/L. Many of the organic UV filters used, as well as antimicrobials and fragrances, are medium to highly hydrophobic chemicals (e.g., log *K*_ow_ >4 for OC, galaxolide, and TCS), with widespread occurrence in sludge^36^ at concentrations of hundreds to thousands of ng/g. According to the data presented here, the annual mass flow of these PCPs toward Swiss WWTPs is between 750 (TCS) and 30,500 kg/year (linoleic acid), which represent a per capita load between 80 and 3400 mg/year/person, respectively. Even higher loads (in the order of several tons per day) have been calculated for surfactants, identified in this work through homologous series analysis, and also used in the formulation of PCPs as well as for household and industrial applications.^22^ The information presented here can be used to refine and/or add novel information about the existing data of consumption of chemicals in Switzerland. For instance, an in-depth report^37^ for the insect repellent DEET estimates a national consumption of 2 tons/year, of which 85 kg/year (approximately 5% of the total) would be ending up in sludge according to our measurements.

Next to the PCPs mentioned above, a phenoxybutyric herbicide (4-(4-chloro-2-methylphenoxy)­butanoic acid, or MCPB), an organophosphorus insecticide TP (2-isopropyl-4-methyl-6-hydroxypyrimidine, or IMHP), an artificial sweetener (neotame), and a plasticizer (bisphenol A, or BPA) were also among the top 10 most abundant contaminants detected in sludge. Although not very often reported in WWTPs, both MCBP and IMHP have been measured in Swiss streams^38^ at levels up to 290 ng/L, as well as in sludge samples from Spain^39^ (e.g., 27 ng/g for IMHP). Here, we identified a total of 129 pesticides in sludge, including 36 fungicides, 35 herbicides, 13 insecticides, and 43 TPs, similar numbers to those reported in Swiss agricultural fields^24^ that underwent SNTS. This abundance reflects the high application of pesticides in the EU, with estimations of 300,000 tons per year according to Eurostat. Their occurrence in urban sewage sludge is also attributed to the use of biocides on facades or in household products, washing of textile products, landscaping, and food processing. For example, fresh fruits and other agricultural products can be treated with fungicides (e.g., DMSA, a dichlofluanid metabolite, in Figure 4A) to extend their storage and shelf life, which can then contribute to loads of these pesticides in wastewater.^40^

Also related to the food industry, our findings regarding the high concentration and widespread occurrence of the sweetener neotame (with a mean value of 1800 ng/g and detected in all sludge samples) are novel. Previous studies in Switzerland reported sucralose and acesulfame as the two main artificial sweeteners detected in WWTP sewage. Both compounds were resistant to removal and proposed as chemical markers for domestic wastewater.^41^ Neotame, an aspartame analogue, was not approved for use in the EU until 2010, so its presence in WWTPs might have been overlooked by earlier studies. This sweetener has been recently suggested as a new potential sewage indicator after detection in both aqueous and particulate phases in Chinese WWTPs.^42^ Lastly, here we identified BPA and other analogues through an MS/MS library search (bisphenol S, score = 87%) and homologous series analysis (4,4’-biphenol, bisphenol E, and F). Usually, their monitoring in sludge has been conducted through target analysis over the past decade, with values in Germany and the United States^43^ similar to those reported here (in the order of several hundreds to thousands of ng/g). Safety concerns due to its estrogenic potential have led to use restrictions of BPA. Consequently, concentrations are expected to decline, whereas environmental emissions of related analogues are likely to increase in the future. In that sense, SNTS workflows could prove very useful to identify new analogues and their TPs. Other chemicals widely used in industrial applications and determined in our sludge samples were benzotriazoles (corrosion inhibitors) and 2-naphthalene sulfonic acid (production of dyes and pharmaceuticals). The complete data set of compounds identified and quantified in sludge can be found in Tables S4–S9 in SI.

### Distribution and Fate of Contaminants in WWTP Sludge

Principal component analysis (PCA) of all features in sludge samples and hierarchical cluster analysis (HCA) of the top 100 compounds identified at Level 1 revealed that the spatial distribution of contaminants and other organic chemicals in sludge was not homogeneous across Swiss WWTPs (Figure 5). Three WWTPs (BAS, HER, and, to a lesser extent, LAU) exhibited differing chemical signatures and were clustered apart from the others. Such differences were evident no matter which extraction method was used and/or if all features or just the identified contaminants were considered for statistical analysis (Figures S8–S12). We hypothesize that one of the reasons behind these unique patterns is different chemical loads to WWTPs in some Swiss regions. Such differences have been already observed in previous sampling campaigns on specific contaminants (e.g., PFAS).^29^ HER receives wastewater from a textile finishing company, whereas LAU and BAS are located in two large Swiss cities (Lausanne and Basel). Much higher concentrations of specific compounds were measured at these locations (i.e., caffeine at 10,400 ng/g in BAS vs national mean at 700 ng/g, lidocaine at 1700 ng/g in HER vs 80 ng/g, or paracetamol, only detected in LAU at 350 ng/g), whereas others were considerably low (i.e., neotame <300 ng/g, national mean = 1750 ng/g). BAS, HER, and LAU also showed elevated concentrations for nonylphenol ethoxylates in a previous study, although their values have been steadily decreasing over the last decades due to use regulation in the EU.^22^ Differing features and organic contaminants identified at Level 1 are depicted in volcano plots (Figures S13–S15). Some of them had a relatively low frequency of detection (<30%) but were highly concentrated (>1000 ng/g) in either BAS, HER, and/or LAU and were overlooked in the previous section focused on prevalent compounds all across Switzerland (Figure 4A). Examples include substances no longer approved for use in the EU that can still be detected in the environment,^44^ such as propachlor-OXA (measured at over 40,000 ng/g), a metabolite of the herbicide propachlor, whose use for approval was withdrawn in 2009.

**Figure 5:**
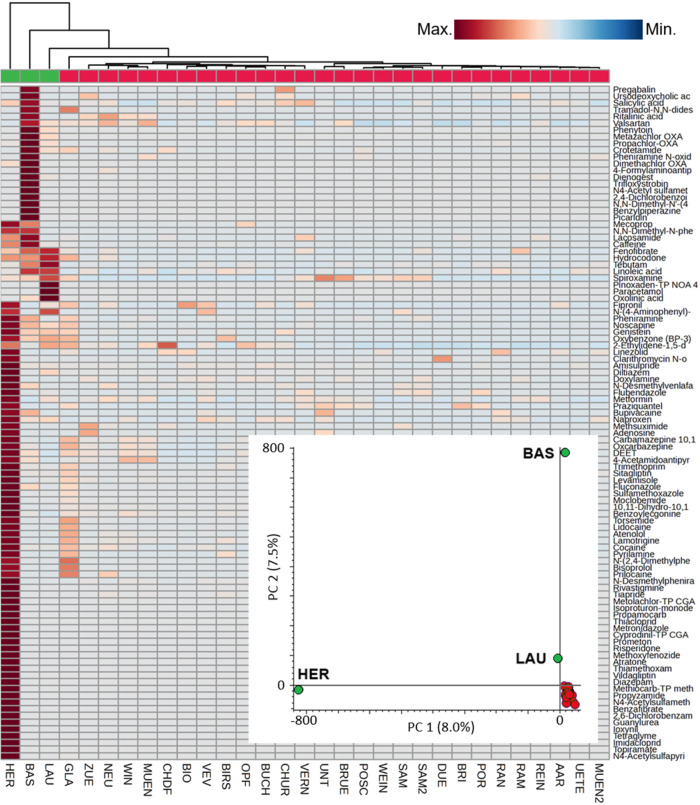
Hierarchical cluster analysis (HCA) showing the distribution of the top 100 organic contaminants (identified at Level 1) in sludge from Swiss WWTPs. Principal component analysis (PCA) using all features detected in sludge is also depicted (embedded). Two main clusters were considered (green = HER, BAS, and LAU WWTPs, red = other WWTPs). The extraction method was MeOH + C18. For other methods, see Figures S11 and S12.

Differences in sludge processing can further explain the different chemical signatures observed. Anaerobic digestion is the preferred option for sewage sludge stabilization in most developed countries, facilitating the conversion of organic matter into biogas and digestate.^8^ This is also the case in Switzerland, where 70% of the sludge is anaerobically digested. The investigated samples were subject to anaerobic digestion for different lengths of time, from 0 (HER) up to 50 days (CHDF) (Table S1). During anaerobic digestion, two counteracting processes affecting contaminant concentration occur:^2^ biodegradation is performed by bacterial communities (thus decreasing the concentration of biodegradable parent compounds), whereas sludge volume is reduced (increasing the concentration and sorption of poorly biodegradable substances and TPs). The combination of such processes with digestion time can lead to varying contaminant patterns in the final sludge. HCA of all features in sludge (Figures S8–S10) show how they clustered in two different groups. The first cluster involved those features that exhibited higher intensities in HER, LAU, and BAS, many of them identified as contaminants at Levels 1 and 2 and already discussed. The second cluster comprised other features with opposing behavior (almost nonexisting in HER, LAU, and BAS but present in other WWTPs). Correlation analysis performed for individual contaminants (Figure 6A) and homologous series (Figure 6B) identified in both clusters showed a substantial relationship of the concentrations/areas of many of them with digestion time (*p*-value <0.05), which could be either positive (*r* > 0.5, compounds accumulated over time) or negative (*r* < −0.5, compounds were removed).

**Figure 6:**
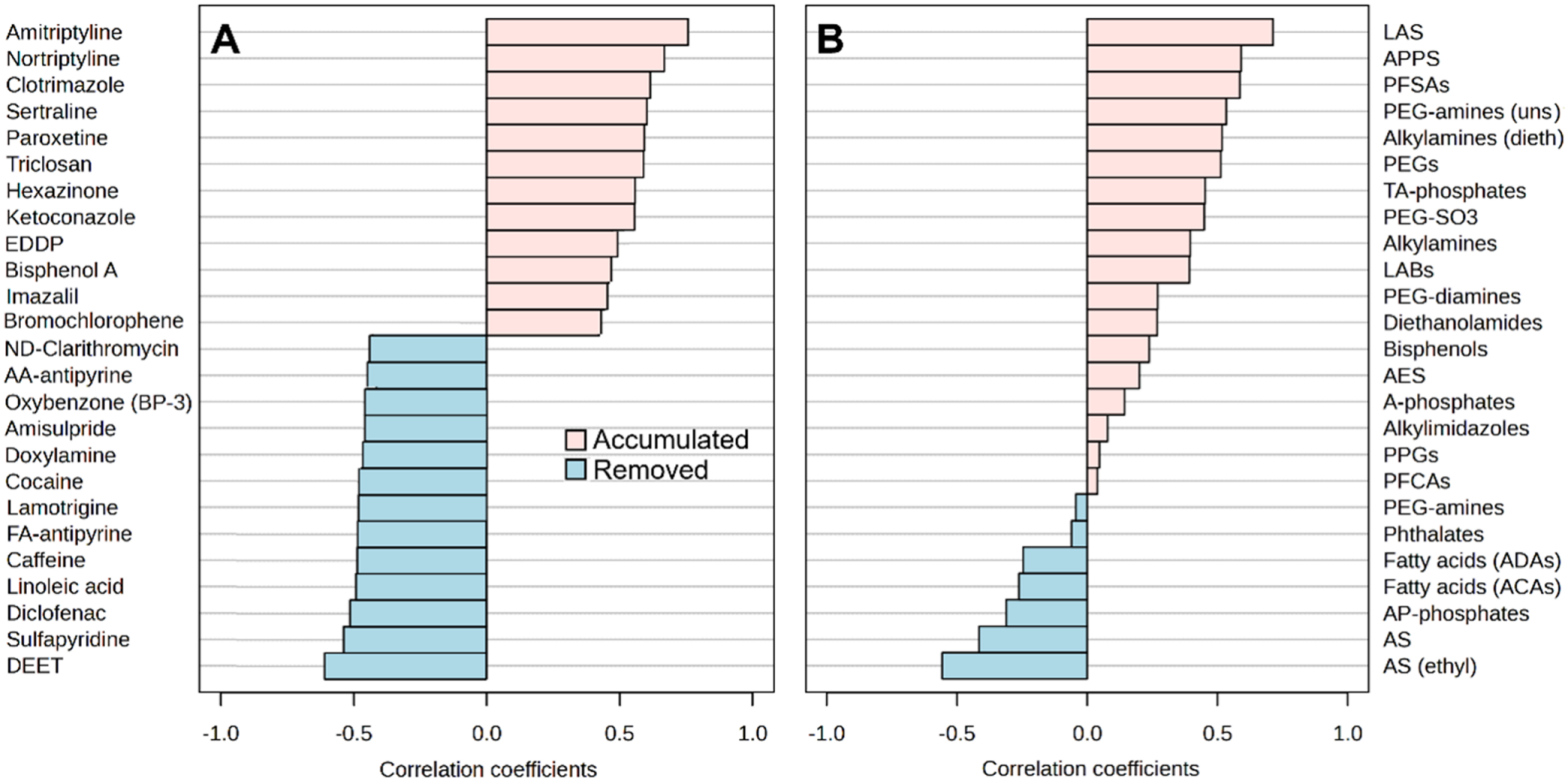
Pearson correlation coefficients (*r*) of concentration of organic contaminants (identified at Level 1) (A) and areas of homologous series (B) with sludge digestion time (from 0 to 50 days).

Among the compounds that exhibited poor biodegradability during anaerobic digestion are well-known cases already documented in the literature,^22,45^ including the anionic surfactant LAS, cationic amine-based surfactants (e.g., alkylamines) and perfluorinated substances (PFSAs). Surfactant transformation products PEGs and PEG-SO_3_ can also increase their concentrations as the result of the degradation of AEOs, alkyl ethoxy sulfates (AES), and ethoxylated amine surfactants, which exhibited negative *r* coefficients. Fatty acids (e.g., linoleic acid), natural products, were also effectively removed during anaerobic digestion as well as caffeine. The attenuation of these and other substances such as DEET and many pharmaceuticals during sludge stabilization has already been observed through specific WWTP surveys and/or laboratory incubations.^8,45^ On the other hand, some plasticizers (BPA), herbicides (hexazinone), antimicrobials (triclosan), antifungals/fungicides (bromochlorophene clotrimazole, imazalil, and ketoconazole), psychiatric drugs (paroxetine, sertraline, amitriptyline, and nortriptyline), and the methadone metabolite EDDP accumulated with anaerobic digestion time in Swiss sludge. These results are in line with previous reports relying on targeted approaches that describe the widespread occurrence and net accumulation of such classes of chemicals in sludge even after anaerobic digestion.^1,7,46,47^

### Environmental Implications

The nationwide survey presented allowed us a glimpse at the large number and quantity of substances used in everyday products for personal and health care as well as in industrial and agricultural chemicals, marketed in an industrialized country. Concentrations of hundreds of organic contaminants reported in sludge through a combination of different and complementary extraction methods followed by semiquantitative analysis after SNTS, are within the same order of magnitude in the great majority of Swiss WWTPs as those reported in previous studies relying on target methods. This showcases the power of new HRMS-based analytical methodologies to simultaneously track prioritized and emerging contaminants and their TPs, as well as to identify (in real time or through retrospective analysis) new chemical threats that may rise over the next decades. Conducting this type of study in sewage sludge is critical due to the millions of tons of biosolids generated every year in the EU and other regions where urban and industrial wastewater treatment is mandatory.^1^ There are already existing guidelines and frameworks limiting the spread of contaminants through biosolids, including EU Directive 91/271 and UNEP 2001, which already establish limits for the concentration of specific contaminants in sewage sludge prior to land application.^2^ The number of regulated contaminants, however, is very low if we consider the numbers and loads presented here, which range from hundreds of grams to thousands of kilograms per year for a myriad of substances that can end up being applied to agricultural soils. The ecotoxicological risks associated with their presence in amended soils are already evident^8^ for some antibiotics (e.g., ciprofloxacin), endocrine-disrupting compounds, and antimicrobials (e.g., TCS). Additional knowledge on the environmental fate and toxicity is required to determine whether or not the observed elevated concentrations of substances that escape regular monitoring programs or are newly introduced may pose any risks to human health and the environment.

The use of national sewage sludge data is also valuable for chemical ranking and prioritization as accumulation of substances in this matrix is indicative of their persistent and bioaccumulative behavior.^4^ Occurrence of contaminants in organisms, including humans, can be extrapolated from information already gathered by sludge monitoring programs. Recent studies have shown that, when compared with chemicals included in biomonitoring as toxic pollutants in humans, there is an overlap of 70–80% between sludge and tissue samples.^4,5^ This agreement in the number and type of contaminants confirms the utility of using noninvasive and cheaper sludge monitoring to estimate the type and average body burden of toxic pollutants in local or national populations. To this end, it would be desirable: (a) to expand the current chemical space covered by LC-ESI-HRMS toward more lipophilic and/or not amenable ESI compounds by modifying LC conditions, adding complementary analytical tools such as GC-HRMS, and/or using alternative ionization sources,^14^ and (b) to establish long-term sludge monitoring programs and apply SNTS to reveal significant changes in emissions/exposure of chemicals in society over time.

## Supplementary Material





## Notes

Additional information on SNTS workflow (Text S1, Figure S2); location of sampled WWTPs (Figure S1); number of features vs retention time (Figure S3); signal suppression/enhancement (Figure S4); Kendrick mass defect plots (Figure S5); correlation heatmap for homologous series (Figure S6); number of organic compounds identified (Figure S7); HCA showing top features and identified compounds (Figures S8–S12); and volcano plots showing differing features and organic compounds (Figures S13–S15) (PDF)
Information on sludge samples (Table S1); lists of reference standards (Tables S2 and S3); information on compounds identified at Level 1 and 2 (Tables S4 and S5); identified homologous series (Table S6); and concentration of top 100 most frequently contaminants in sludge as a function of the extraction method (Tables S7–S9) (XLSX)
