# Supplementary material for: Suspect and Nontarget Screening of Organic Micropollutants in Swiss Sewage Sludge: A Nationwide Survey
Source: Environ Sci Technol. 2025 Apr 8;59(15):7688–98. doi: 10.1021/acs.est.4c13217 (PMC12444985; doi:10.1021/acs.est.4c13217)
Supplement: Supplementary file 1 [file es4c13217_si_001.pdf]

Title:

Suspect and nontarget screening of organic micropollutants in Swiss sewage sludge: a nationwide survey

Authors:

Pablo A. Lara-Martín[1*], Lena Schinkel[2], Yves Eberhard[2], Walter Giger[3], Michael Berg[2], Juliane Hollender[2,4]

[1] Department of Physical Chemistry, Faculty of Marine and Environmental Sciences, University of Cadiz, Puerto Real, 11510, Spain

[2] Eawag, Swiss Federal Institute of Aquatic Science and Technology, 8600 Dübendorf, Switzerland

[3] Giger Research Consulting, 8049 Zurich, Switzerland

[4] Institute of Biogeochemistry and Pollutant Dynamics (IBP), ETH Zurich, 8092 Zurich, Switzerland

[*] Corresponding author's e-mail: pablo.lara@uca.es

Number of pages: 18

Number of figures: 15

Number of tables: 9 (in Excel file)

Text S1. Suspect and nontarget screening workflow description

Figure S2 shows the diagram for the suspect and nontarget screening (SNTS) workflow developed for identification of compounds in Swiss sewage sludge. Compound Discoverer 3.3 gathers all mass spectrometric (MS) signals and combine them into features (defined in the software as 'Compounds'), which include isotope and adduct grouping. The workflow used here was adapted from Lara-Martín et al. (2022) and is composed by several nodes with the following settings:

Select Spectra: Lower RT limit = 0.5 min, Polarity Mode -/+.

Align Retention Times: Alignment Model = Adaptive curve, Maximum Shift = 0.5 min, Mass Tolerance = 5 ppm.

Detect Compounds: Ions = M+H, M-H, M+2H, M-2H, 2M+H, 2M-H, M+Na, M+NH4, Intensity Tolerance = 30, Mass Tolerance = 5 ppm, Max. Element Counts = C90 H190 Br3 Cl4 K2 N10 Na2 O18 P3 S5, Min. Element Counts = C H, Min. Peak Intensity = 50000, S/N Threshold = 3.

Group Compounds: Mass Tolerance = 5 ppm, RT Tolerance = 0.1 min, Minimum Valley = 10%, Align Peaks = False, Preferred Ions = M+H, M-H, Area Integration = Most Common Ion.

Mark Background: Max. Sample/Blank = 5, Max. Blank/Sample = 0, Hide Background = True.

Assign Compound Annotations: Mass Tolerance = 5 ppm, Data Sources = #1 mzCloud Search, #2 = mzVault (MassBank EU) Search, #3 MassList (NORMAN Substance Dataset) Search, #4 ChemSpider Search, #5 Predicted Compositions, Scoring Rules = Use mzLogic = True, Use Spectral Distance = True, SFit Threshold = 20, SFit Range = 20.

Search mzCloud: Compounds Classes = All, Library = Autoprocessed; Reference, Search MSn Tree = False, Identity Search for DDA search = Cosine, Match Activation Type = True, Match Activation Energy = Match with Tolerance, Activation Energy = 50, Apply Intensity Threshold = True, Similarity Search = None, Match Factor Threshold = 70.

Search mzVault: mzVault Library = MassBank EU, Compounds Classes = All, Match Ion Activation Type = True, Match Ion Activation Energy = Any, Ion Activation Energy Tolerance = 200, Match Ionization Method = False, Apply Intensity Threshold = True, Precursor Mass Tolerance = 10 ppm, Match Analyzer Type = False, Search Algorithm = HighChem HighRes, Match Factor Threshold = 70.

Search Mass Lists: Mass Lists = NORMAN Substance Database 2022, Eawag Reference Standard Custom List (Tables S2 and S3), Use Retention Time = True; RT Tolerance = 0.25 min, Search Mode = By Formula or Mass, Mass Tolerance = 5 ppm.

Search ChemSpider: Databases = ACToR, DrugBank, Eawag, EPA, and FDA, Search Mode = By Formula or Mass, Mass Tolerance = 5 ppm, Max no. of results per compound = 20, Max. no. of predicted compositions to be searched = 3.

Predict Compositions: Mass Tolerance = 5 ppm, Min. Element Counts = C H, Max. Element Counts = C90 H190 Br3 Cl8 F18 N10 O18 P3 S5, Min. RBDE = 0, Max. RBDE = 40, Min. H/C = 0.1, Max. H/C = 3.5, Max. no. candidates = 10, Intensity Tolerance = 30 %, Intensity Threshold = 0.1 %, S/N Threshold = 3, Use Dynamic Recalibration = True, Use Fragment Matching = True.

Calculate Mass Defect: Fractional Mass = False, Standard Mass Defect = False, Relative Mass Defect = False, Kendrick Mass Defect = True, Kendrick Formula = CH2, CF2, C2H4, C3H6, C2H4O, and C3H6O.

Apply mzLogic: Max. no. compounds = 0, Max no. mzCloud Similarity Results = 10, Match Factor Threshold = 30.

Apply Spectral Distance: Mass Tolerance = 5 ppm, Intensity Tolerance = 30%, Intensity Threshold = 0.1%, S/N Threshold = 3, Use Dynamic Recalculation = True.

Differential Analysis: Log10 Transformation = True, Update Peak Rating = True, Area Contribution = 3, CV Contribution = 10, FWHM to Base Contribution = 5, Jaggedness Contribution = 5, Modality Contribution = 5, Zig-Zag Index Contribution = 5.

After processing, identification of compounds was performed following the guidelines established by Schymanski et al. (2014): Level 1 = compound identity confirmed by reference standard, Level 2 = probable structure by library spectrum match, Level 3 = tentative candidate by a combination of suspect mass list search, in-silico fragmentation match with mass spectrum, and/or homologous series annotation, and Level 4 = unequivocal molecular formula. Compound annotations and areas were then exported to Excel files for quantification and statistical analysis. Quantification was achieved by constructing calibration curves with analytical standards which were injected under the same conditions than the samples at 5 concentration levels (0.1, 1, 10, 100 and 1000 µg/L). When possible, concentrations were corrected by considering the ratio of the internal standard areas in the calibration curves and in the samples. Statistical analyses were performed through MetaboAnalyst 6.0 (www.metaboanalyst.ca) and included: 2D Scores Plot (PC1 and PC2) from Principal Component Analysis (PCA), Volcano Plot (p-value threshold = 0.1), Correlation Heatmaps (Distance measure = Pearson r, p-value threshold = 0.1), and Hierarchical Clustering Heatmaps (Distance measure = Euclidean, Clustering method = Ward).

References

Lara-Martín, P.A.; Chiaia-Hernández, A.C.; Biel-Maeso, M.; Baena-Nogueras, R.M.; Hollender, J. Tracing urban wastewater contaminants into the Atlantic Ocean by nontarget screening. *Environ. Sci. Technol.* **2020**, *54*, 3996-4005.

Schymanski, E.L.; Jeon, J.; Gulde, R.; Fenner, K.; Ruff, M.; Hollender, J. Identifying Small Molecules via High Resolution Mass Spectrometry: Communicating Confidence. *Environ. Sci. Technol.* **2014**, *48*, 2097-2098.

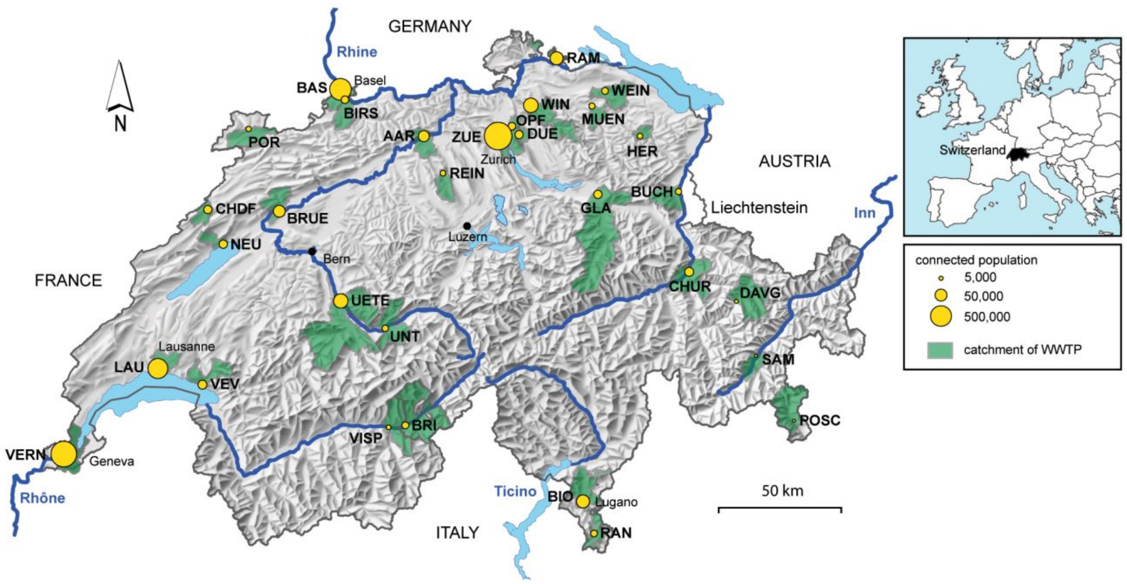

Figure S1. Location of the sampled WWTPs in Switzerland, their connected population and catchment.

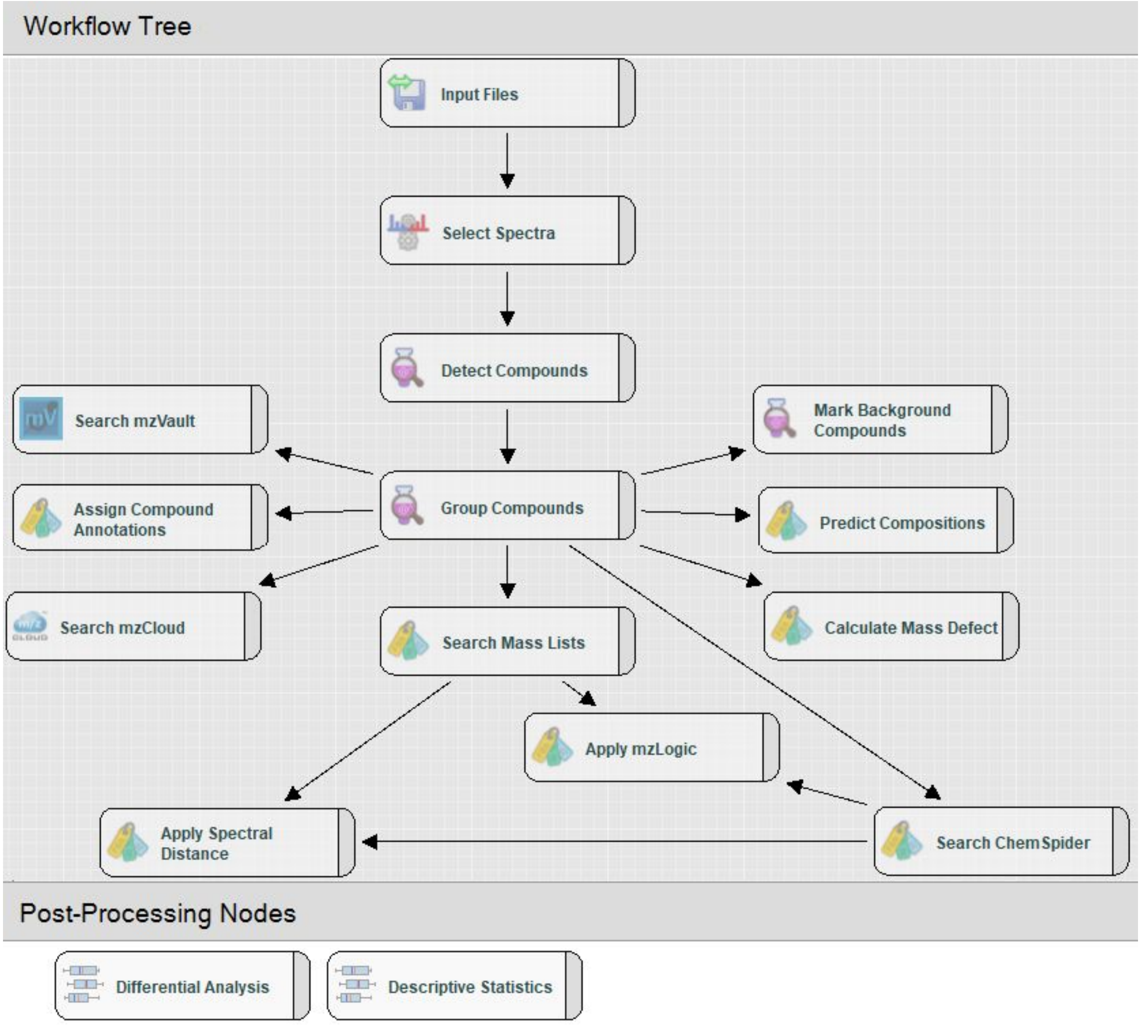

Figure S2. Workflow showing the different nodes used in Compound Discoverer 3.3 to identify features in sewage sludge samples.

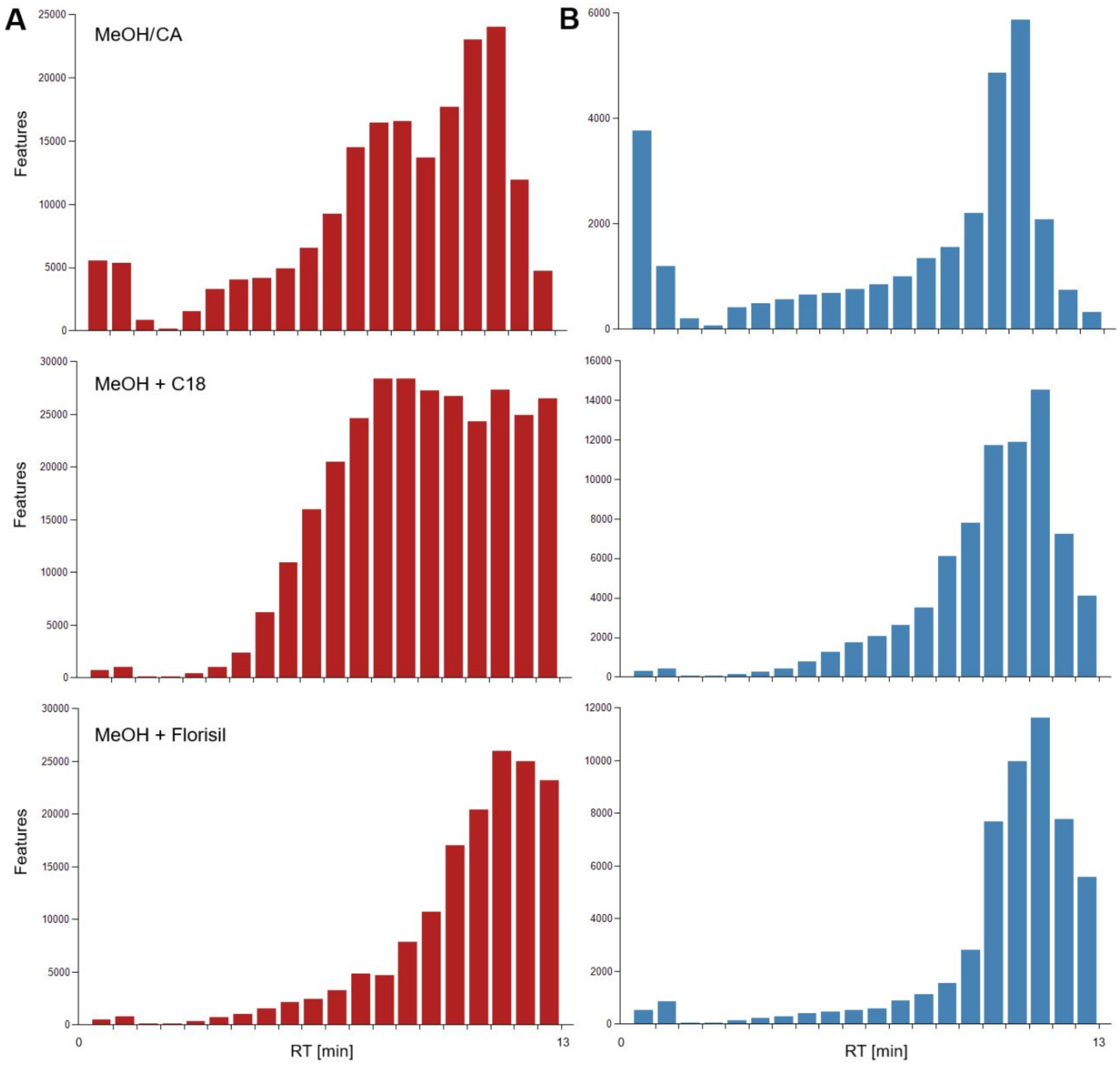

Figure S3. Number of features extracted by the three different methods used and detected under A) ESI positive ionization mode and B) ESI negative ionization mode in sewage sludge as a function of their chromatographic retention time.

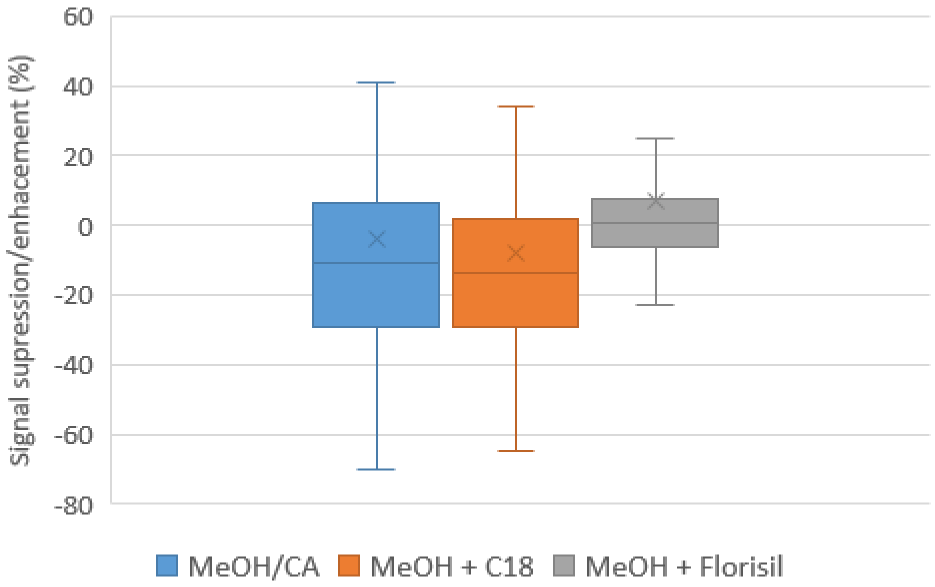

Figure S4. Signal suppression (-) / enhancement (+) for internal standards (see Table S2 for the full list of compounds) spiked to sewage sludge samples extracted using the three different methods.

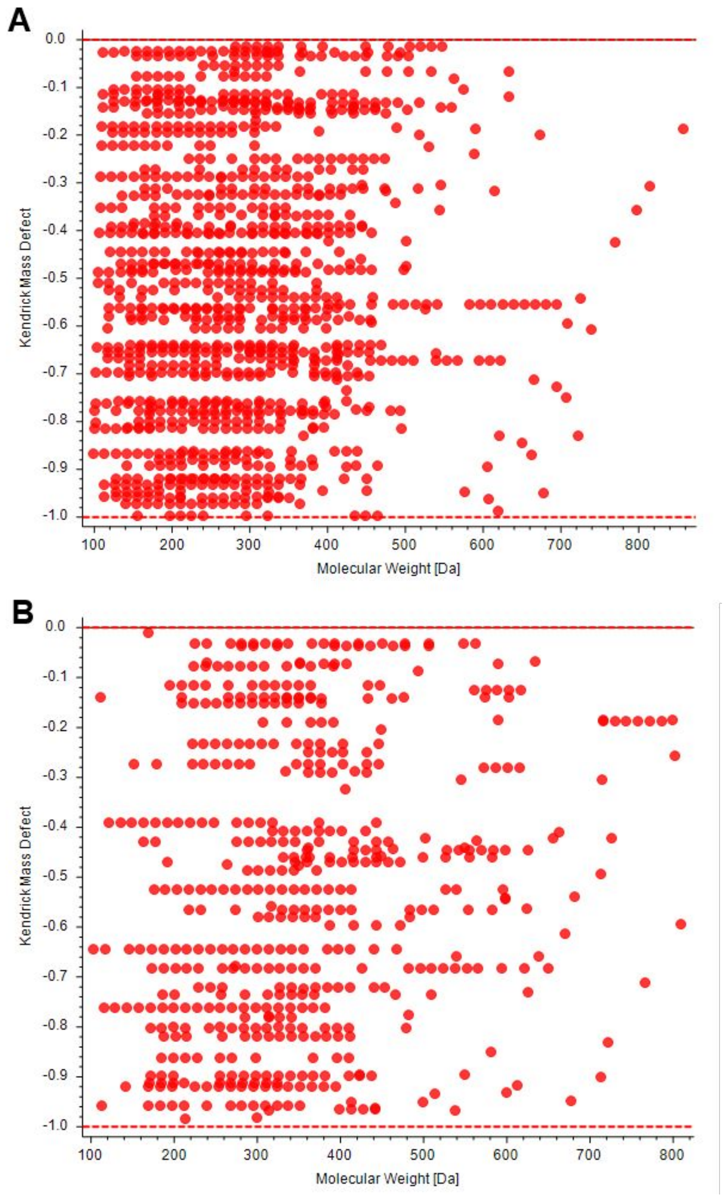

Figure S5. Kendrick mass defect plots (repeating unit CH_2) showing the homologous series detected in sewage sludge samples under A) ESI positive ionization mode and B) ESI negative ionization mode.

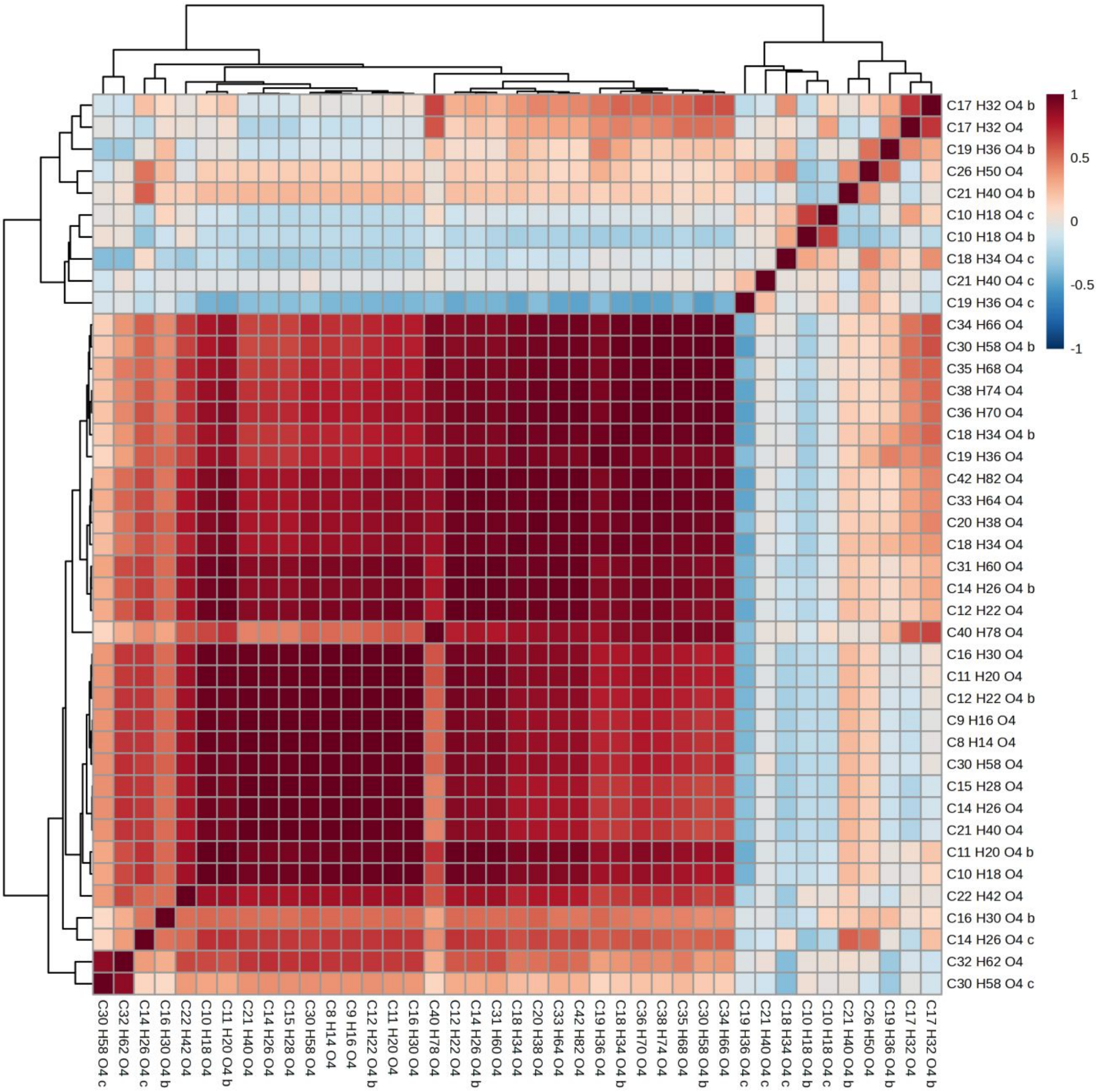

Figure S6. Pearson correlation heatmap for the homologous series C8 H14 O4 to C42 H82 O4 (tentatively identified as alkyl dicarboxylic acids).

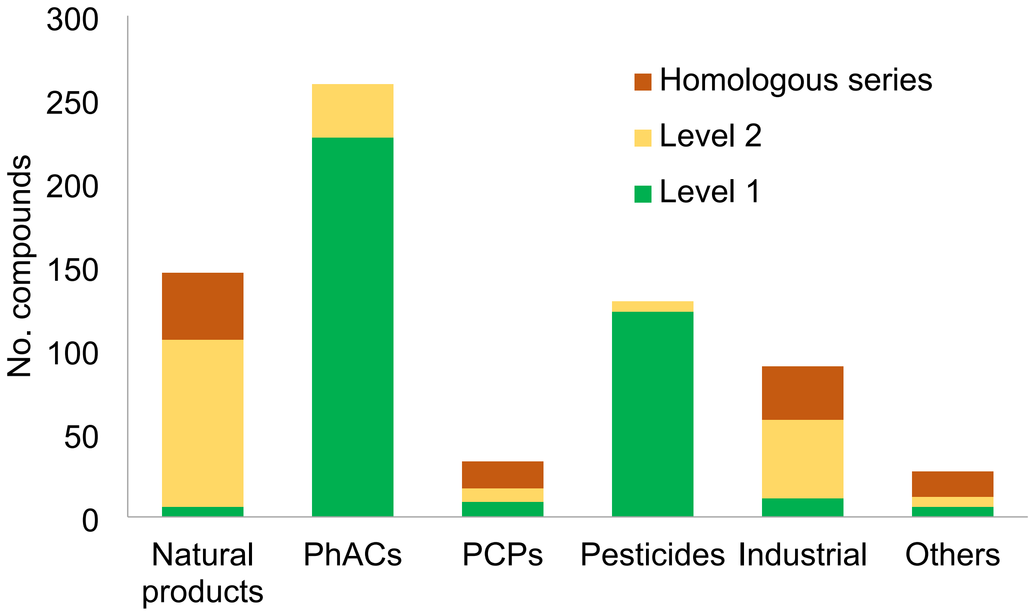

Figure S7. Number of organic compounds identified in sewage sludge samples at Level 1 and 2, as well as homologous series, sorted by chemical class.

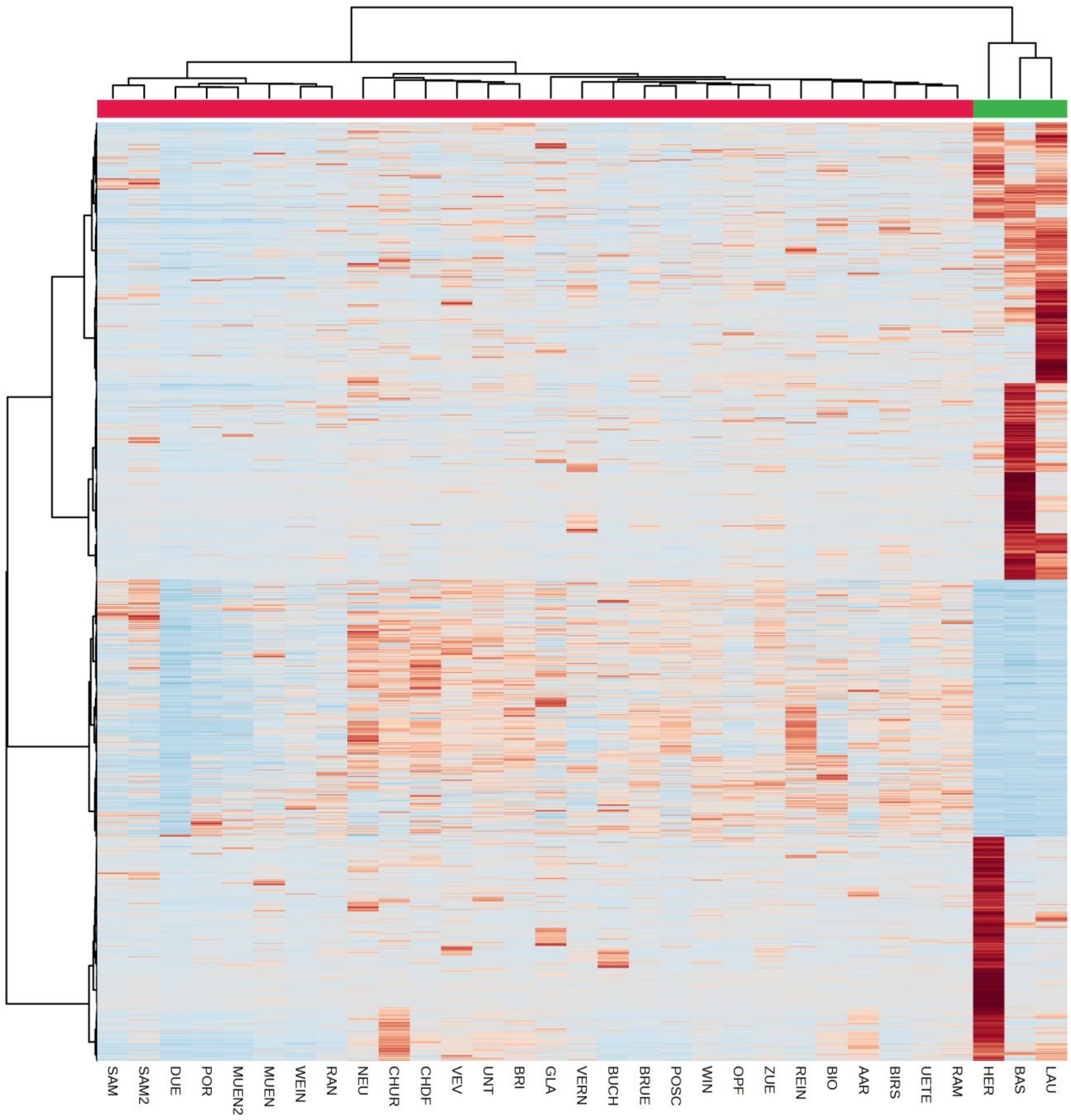

Figure S8. Hierarchical cluster analysis showing top 10000 features in sewage sludge samples from different Swiss WWTPs (extracted using method MeOH + C18).

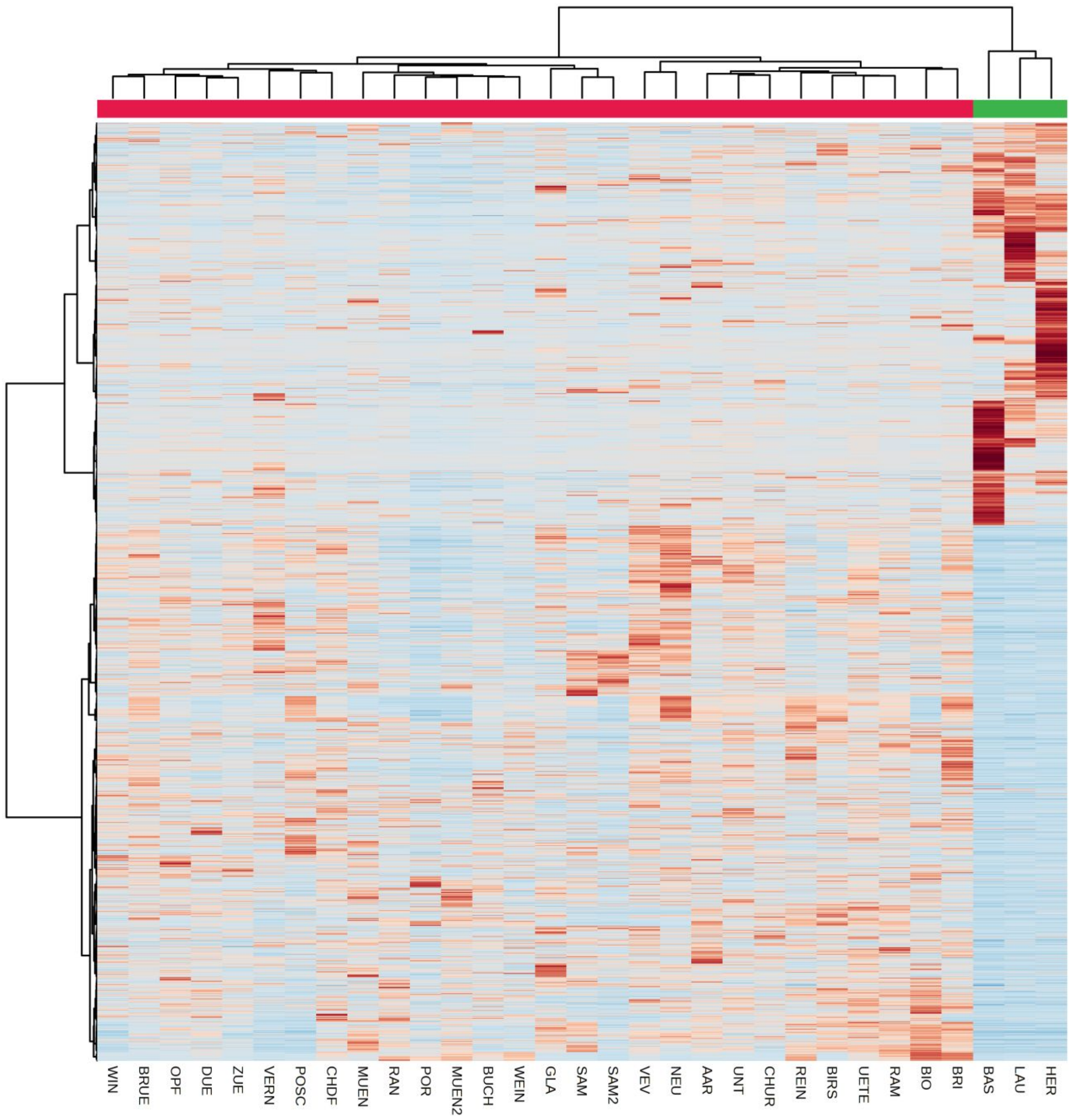

Figure S9. Hierarchical cluster analysis showing top 10000 features in sewage sludge samples from different Swiss WWTPs (extracted using method MeOH + Florisil).

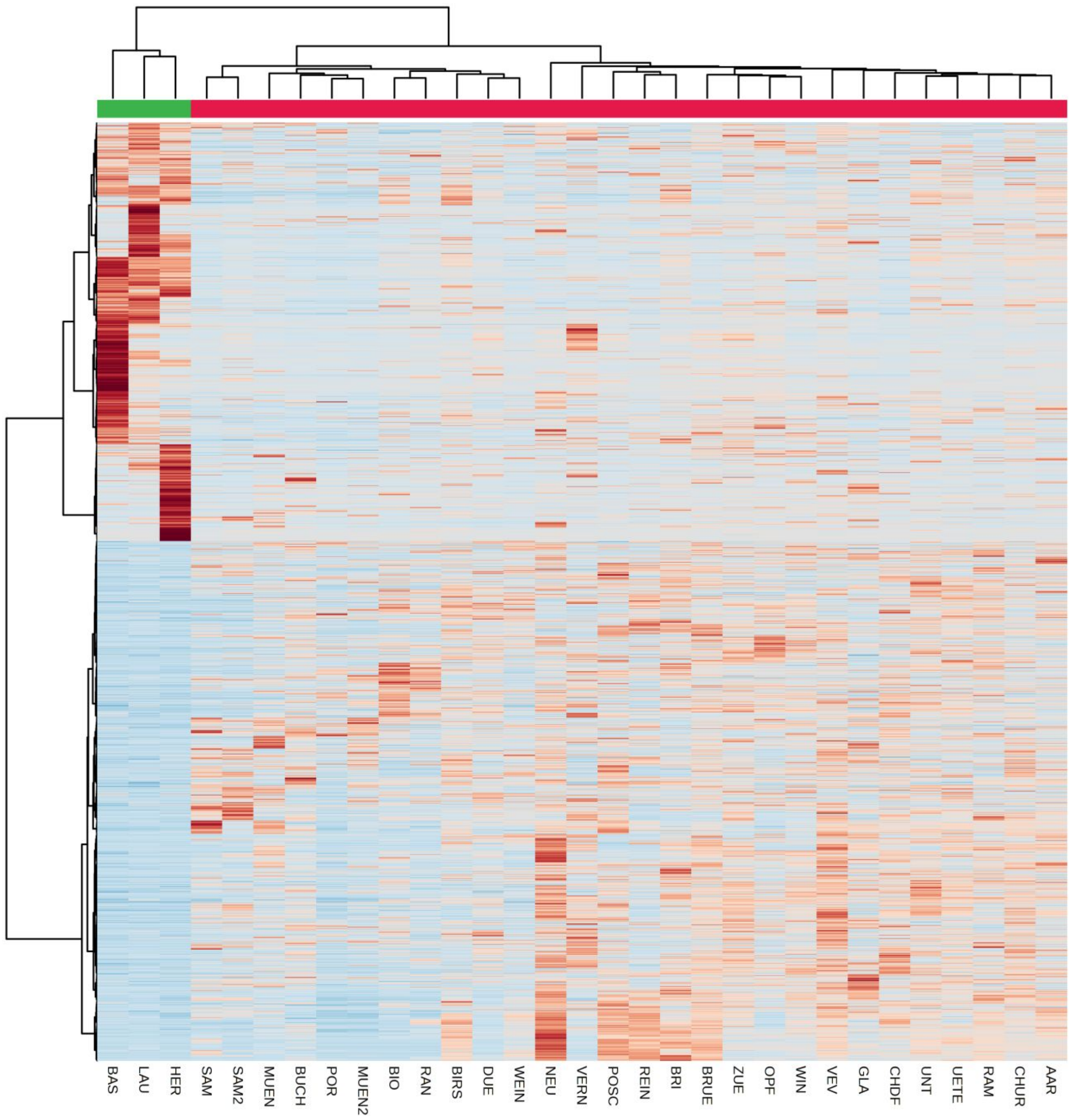

Figure S10. Hierarchical cluster analysis showing top 10000 features in sewage sludge samples from different Swiss WWTPs (extracted using method MeOH/CA).

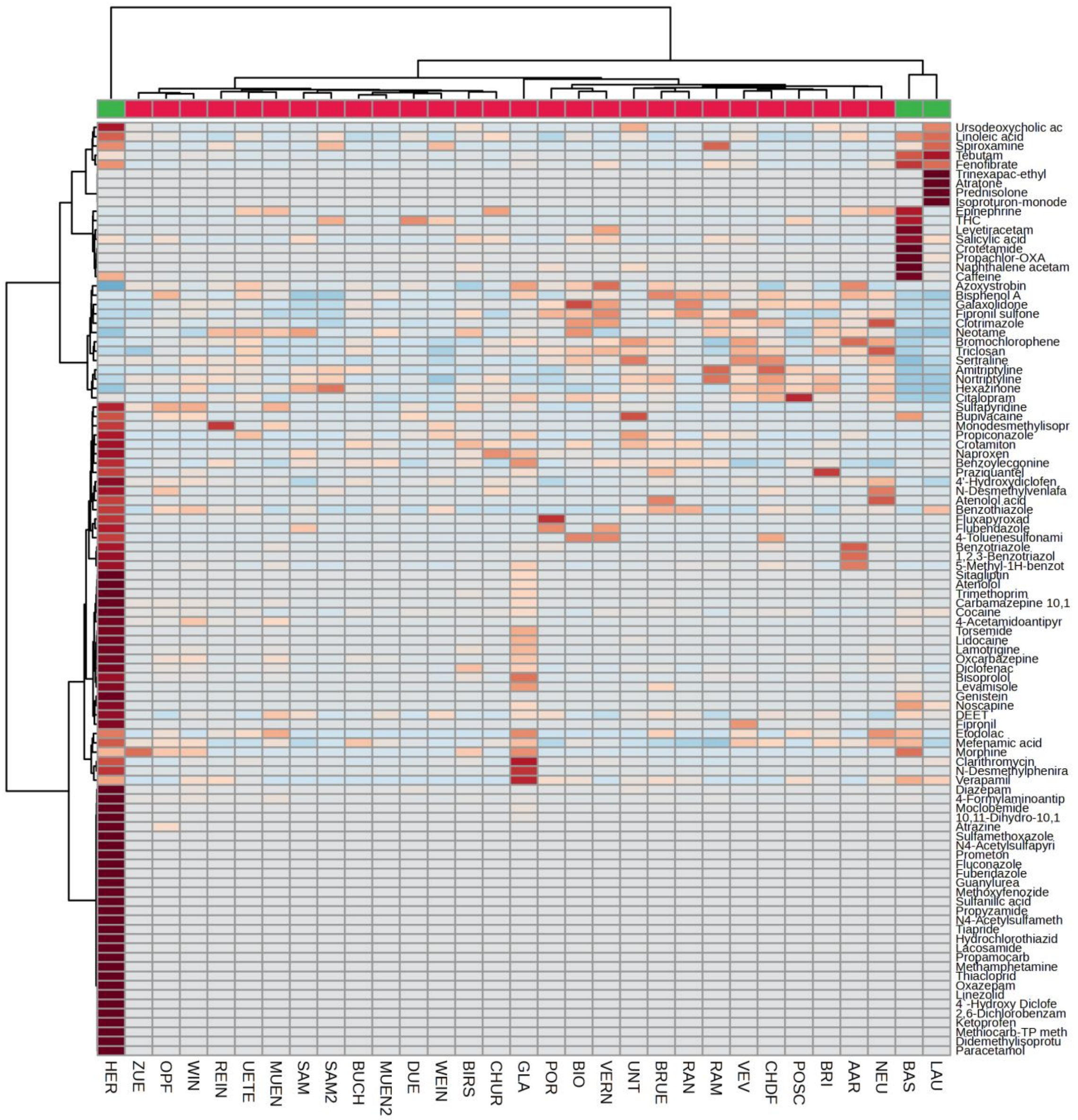

Figure S11. Hierarchical cluster analysis showing top 100 organic contaminants (identified at Level 1) in sewage sludge samples from different Swiss WWTPs (extracted using method MeOH + Florisil).

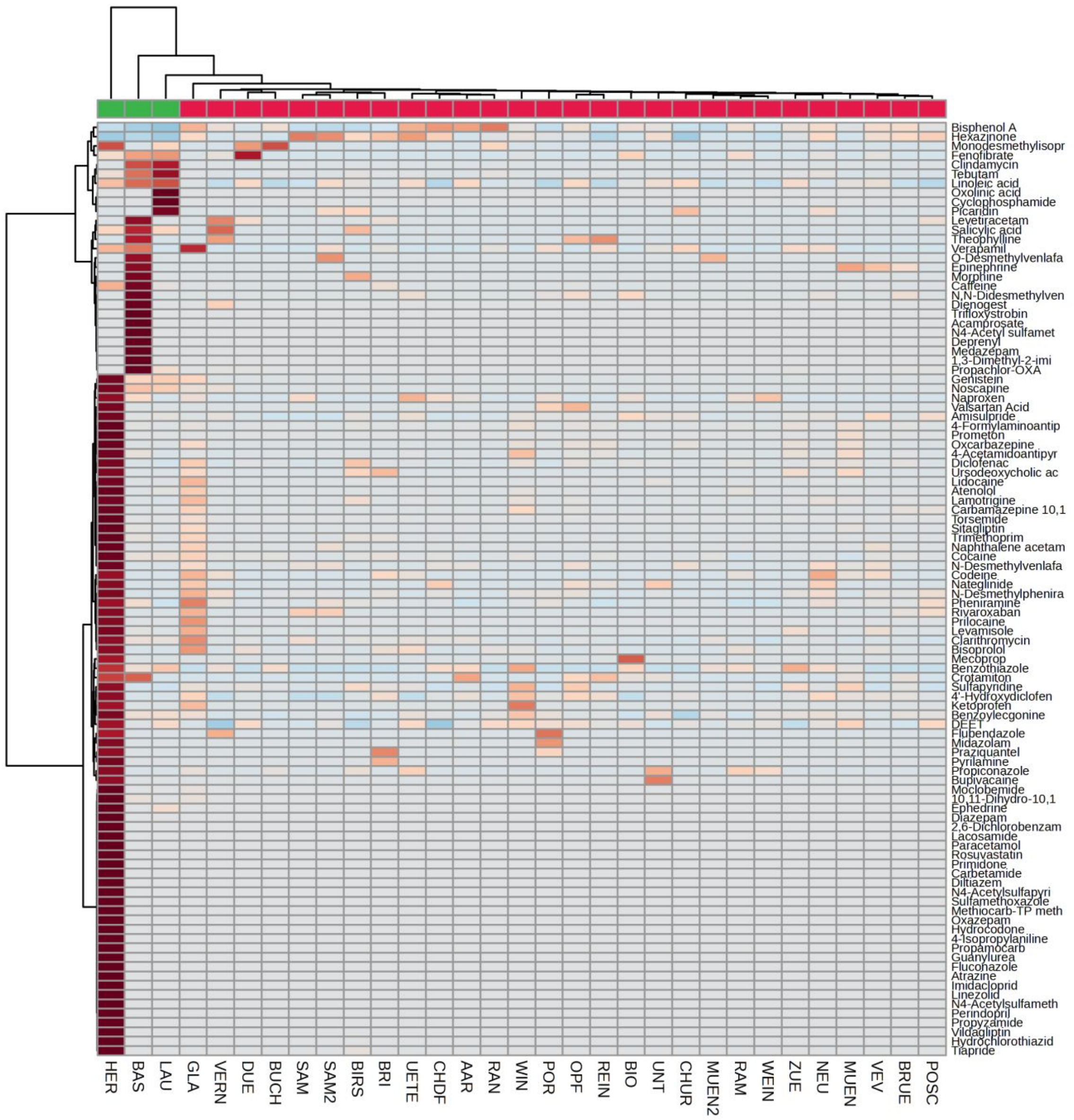

Figure S12. Hierarchical cluster analysis showing top 100 organic contaminants (identified at Level 1) in sewage sludge samples from different Swiss WWTPs (extracted using method MeOH/CA).

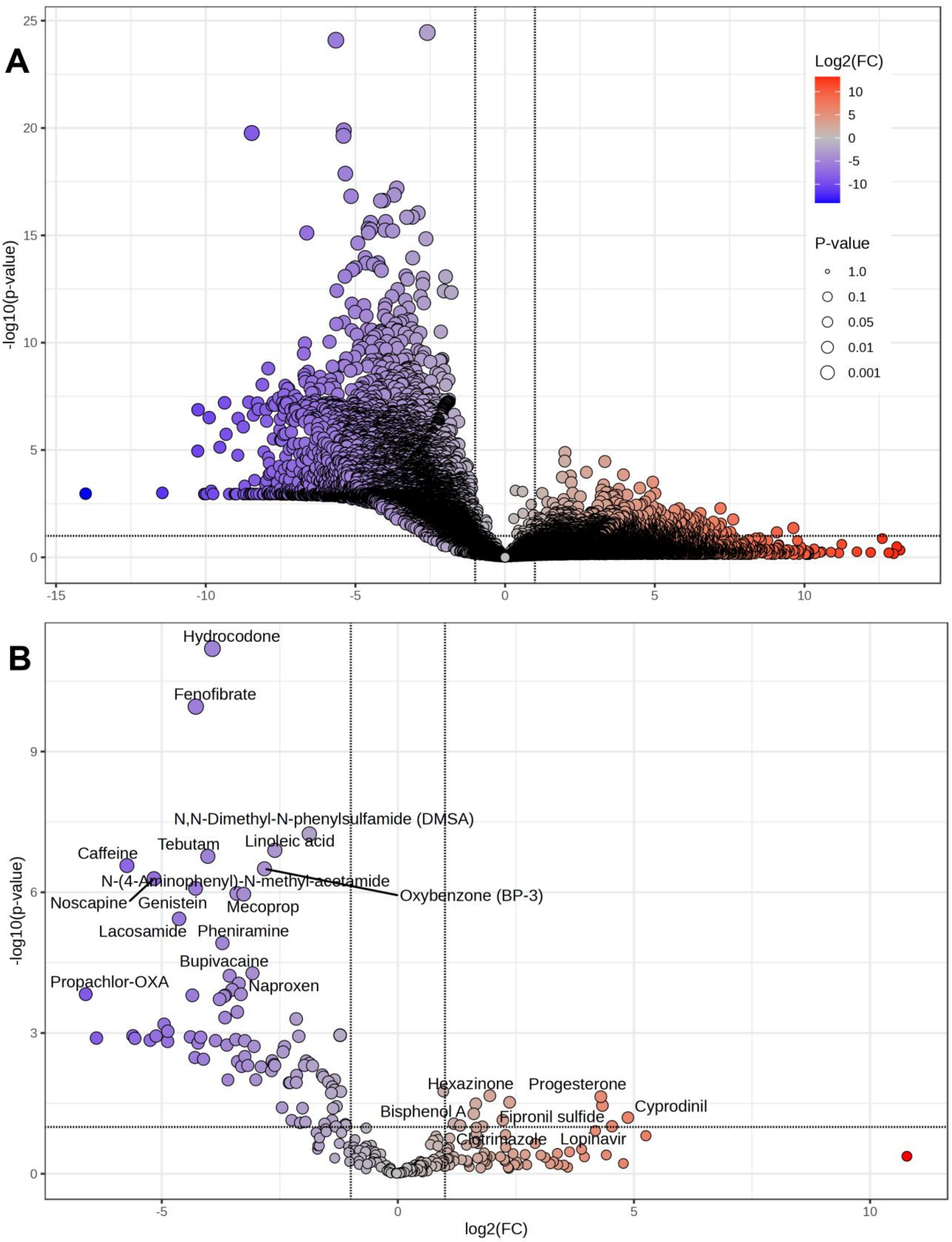

Figure S13. Volcano plots showing differing features (A) and organic compounds identified at Level 1 (B) between sludge samples from BAS, LAU, and HER and other Swiss WWTPs (extracted using method MeOH + C18).

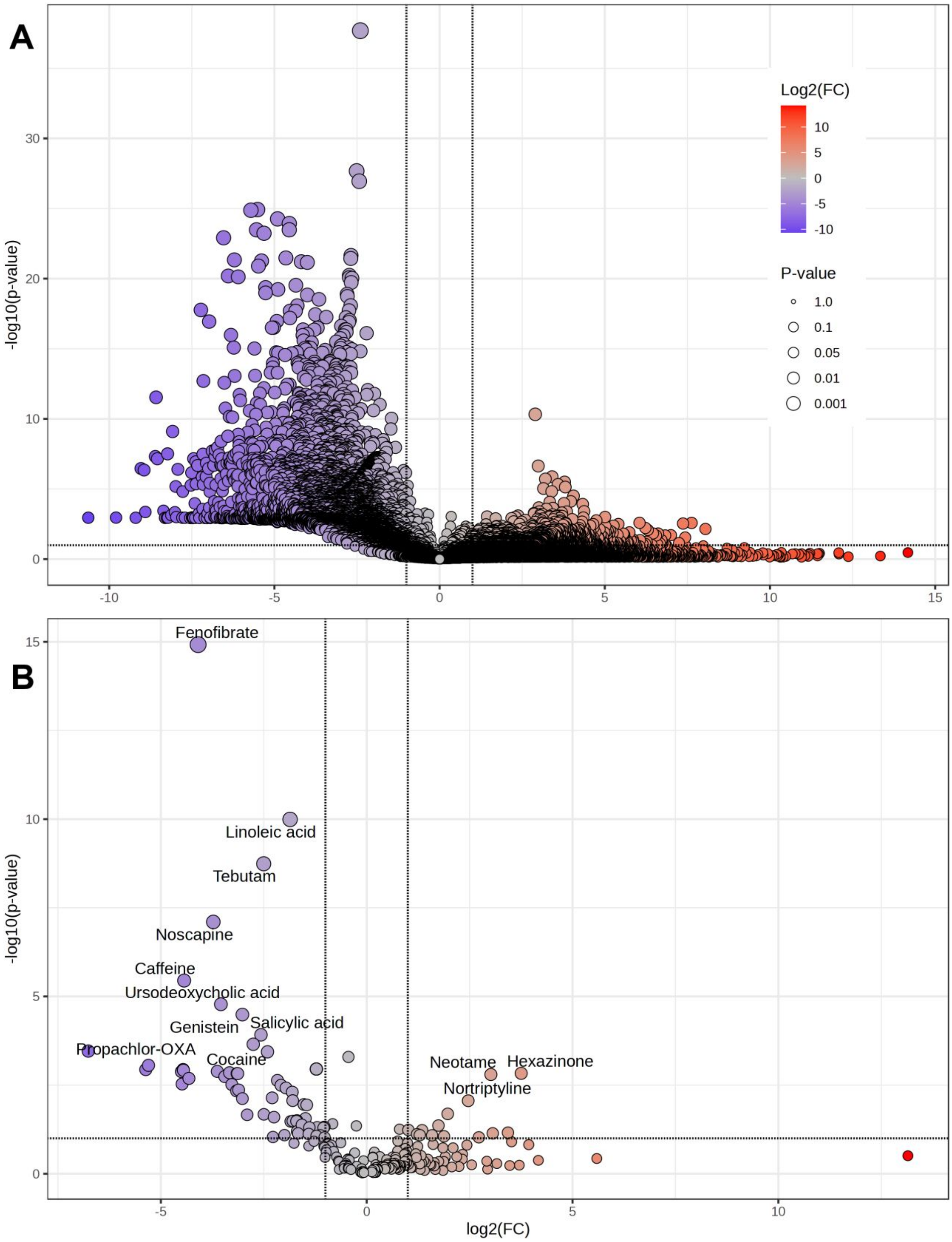

Figure S14. Volcano plots showing differing features (A) and organic compounds identified at Level 1 (B) between sludge samples from BAS, LAU, and HER and other Swiss WWTPs (extracted using method MeOH + Florisil).

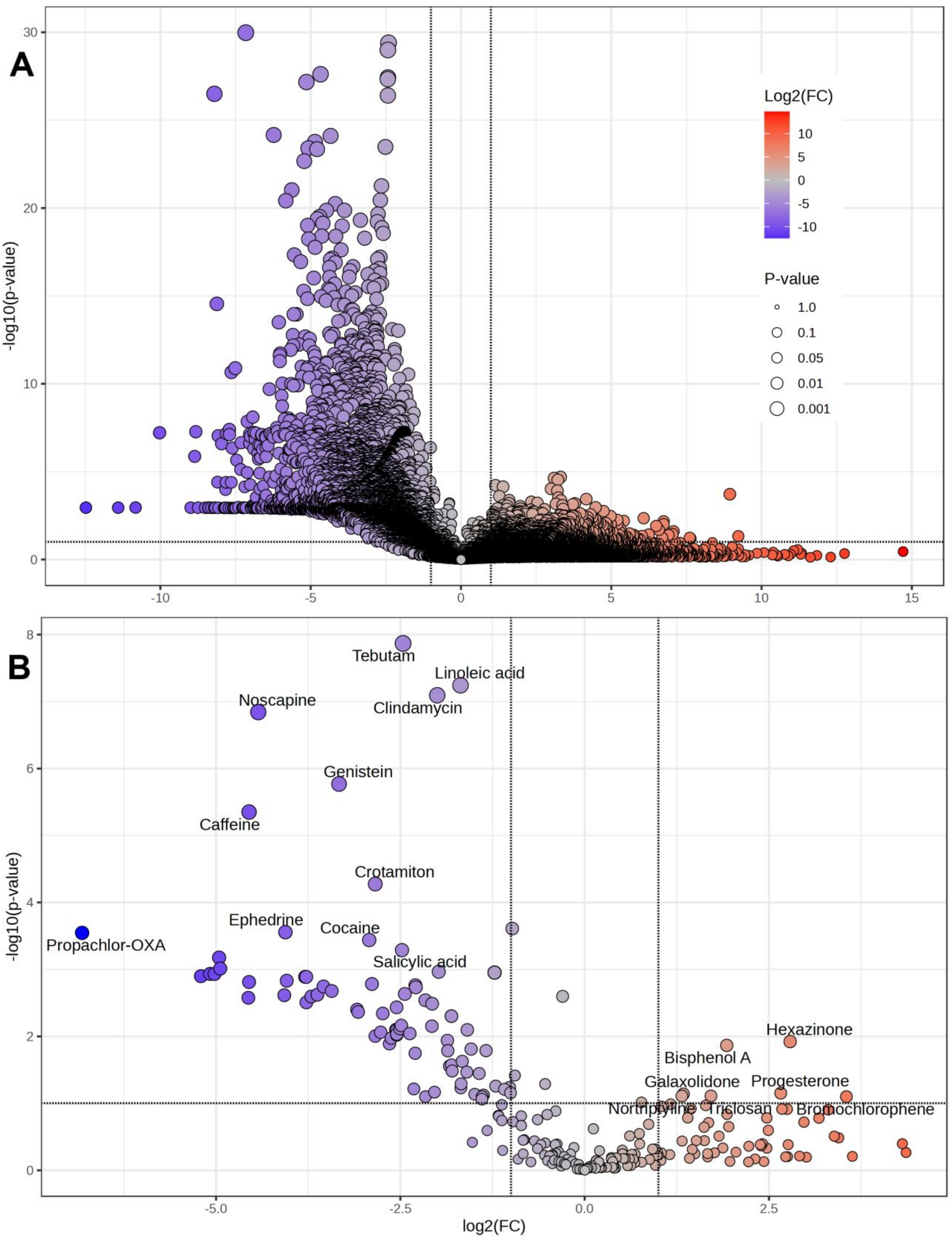

Figure S15. Volcano plots showing differing features (A) and organic compounds identified at Level 1 (B) between sludge samples from BAS, LAU, and HER and other Swiss WWTPs (extracted using method MeOH/CA).
